# Reconstruction of the Evolutionary History of *Saccharomyces cerevisiae* x *S. kudriavzevii* Hybrids Based on Multilocus Sequence Analysis

**DOI:** 10.1371/journal.pone.0045527

**Published:** 2012-09-25

**Authors:** David Peris, Christian A. Lopes, Armando Arias, Eladio Barrio

**Affiliations:** 1 Institut “Cavanilles” de Biodiversitat i Biologia Evolutiva, Universitat de València, València, Spain; 2 Departamento de Biotecnología, Instituto de Agroquímica y Tecnología de Alimentos, CSIC, València, Spain; 3 Grupo de Biodiversidad y Biotecnología de Levaduras, Instituto Multidisciplinario de Investigación y Desarrollo de la Patagonia Norte (CONICET-UNCo), Facultades de Ingeniería y Ciencias Agrarias, Universidad Nacional del Comahue, Neuquén, Argentina; 4 Laboratorio de Biotecnología, Unidad de Botánica y Zoología, CUCBA, Universidad de Guadalajara, Zapopan, Jalisco, Mexico; 5 Departamento de Genètica, Universitat de València, València, Spain; University of Toronto, Canada

## Abstract

In recent years, interspecific hybridization and introgression are increasingly recognized as significant events in the evolution of *Saccharomyces* yeasts. These mechanisms have probably been involved in the origin of novel yeast genotypes and phenotypes, which in due course were to colonize and predominate in the new fermentative environments created by human manipulation. The particular conditions in which hybrids arose are still unknown, as well as the number of possible hybridization events that generated the whole set of natural hybrids described in the literature during recent years. In this study, we could infer at least six different hybridization events that originated a set of 26 *S. cerevisiae* x *S. kudriavzevii* hybrids isolated from both fermentative and non-fermentative environments. Different wine *S. cerevisiae* strains and European *S. kudriavzevii* strains were probably involved in the hybridization events according to gene sequence information, as well as from previous data on their genome composition and ploidy. Finally, we postulate that these hybrids may have originated after the introduction of vine growing and winemaking practices by the Romans to the present Northern vine-growing limits and spread during the expansion of improved viticulture and enology practices that occurred during the Late Middle Ages.

## Introduction

The first evidence of production of fermented beverages dates back to 7000 BC in the Neolithic village of Jiahu in China [1], but the earliest evidence of winemaking is traced to Iran at the Hajji Firuz Tepe site (5400-5000 BC) [2]. From these origins in the slopes of northern Zagros, eastern Taurus and Caucasus Mountains, vineyards and grape wine production gradually spread to adjacent regions of the Fertile Crescent such as Mesopotamia and the Jordan Valley, and beyond, to the Eastern Mediterranean regions of Egypt, Phoenicia, Crete and Greece (5000 BC). Colonization by the Phoenicians, Carthaginians and Greek spread winemaking far across the Western Mediterranean regions of Southern Europe and Northern Africa. By 500 BC, wine was being produced in Italy, Sicily, Southern France, the Iberian Peninsula and the Maghreb. Vine cultivation was later extended by the Romans to the Northern limits of their empire (100 BC-100 AD). The next important expansion of winemaking was during the European colonization of America (16^th^ century), South Africa (17^th^ century), and Australia and New Zealand (18–19^th^ centuries) [3], [4].

On the other hand, beer elaboration is first recorded in the Mesopotamian region and in Egypt. Brewing diverged into two processes mainly differentiated by the prevailing fermentation temperature: ale, acquired from the Middle East by Germanic and Celtic tribes around the 1st century AD, and lager, which appeared during the Late Middle Ages in Europe [5], [6].

A fortuitous domestication that acted on the *S. cerevisiae* populations is associated with wine and beer elaboration: it occurred as a consequence of the expansion of these fermentation processes. The first genetic diversity characterization of *S. cerevisiae* strains, isolated from different sources, showed clear differences between wild and domesticated strains [7]. Another study [8] evaluated the genetic variability of ∼250 *S. cerevisiae* strains based on four nuclear gene sequences, and revealed for some genes the presence of two groups of alleles that differentiated wine strains from those isolated from other, non-wine, sources. Liti et al. [9] performed a genetic-population analysis based on whole genome sequences of 36 *S. cerevisiae* strains and reported the presence of five ‘clean’ (pure) lineages and different ‘mosaic’ (recombinant) strains. One of the ‘clean’ genotypic lineages comprises a number of wine strains from different geographic origins as well as European non-wine strains, and therefore, it was called wine/European population, the other lineages corresponded to strains isolated from other sources and origins [9].

In recent years, hybrids between *S. cerevisiae* and other *Saccharomyces* species such as the cryotolerant *S. uvarum* [10]–[12] and *S. kudriavzevii* [13]–[17] have been isolated from wine, cider and brewing fermentations, and other sources. These discoveries suggest that hybridization between different *Saccharomyces* species has been a frequent phenomenon in their evolution, particularly relevant during the adaption of *Saccharomyces* to fermentative conditions [18]–[20]. Some hybrids can be predominant even in the most Northern winemaking regions from Europe, very likely due to a better adaptation to growth at lower temperatures acquired from the non-*cerevisiae* parental, compared to *S. cerevisiae* [15], [18], [19], [21].

Some reports carried out on a set of wine and beer *S. cerevisiae* x *S. kudriavzevii* hybrid strains suggested that those hybrids could be generated from hybridization between wine strains of *S. cerevisiae* and natural European strains of *S. kudriavzevii*; however, those results were not completely conclusive [17], [22], [23]. The aim of this study was to evaluate, by means of a multigenic sequence approach, the potential origin of 24 *S. cerevisiae* x *S. kudriavzevii* and 2 *S. cerevisiae* x *S. kudriavzevii* x *S. uvarum* hybrid strains obtained from wine, beer and two other non-fermentative sources. The possible number of hybridization events that gave origin to the complete set of hybrids was also proposed based on the results obtained in this work and in previously reported data.

## Methods

### *Saccharomyces* Strains, Culture Media and Nucleotide Sequences

Twenty-six *S. cerevisiae* x *S. kudriavzevii* hybrid strains from different origins (Table S1) and seven strains belonging to *S. kudriavzevii* species (Table S2) were used in this study. Yeasts were grown at 28°C in GPY medium (2% glucose, 0.5% peptone, 0.5% yeast extract).

Nucleotide sequences corresponding to representative *S. cerevisiae* wine and non-wine alleles according to Arias [8] for genes *BRE5*, *CAT8*, *EGT2* and *GAL4* were also included in this study (Table S3 and Table S4).

Sequences for genes *BRE5*, *CAT8*, *CYC3*, *CYR1*, *EGT2*, *CAT8*, *GAL4* and *MET6* from *S. cerevisiae* strains (Table S2) representative of each pure population defined by Liti *et al.* [9] were obtained from SGRP (*Saccharomyces* Genome Resequencing Project, version 2 assemblies (20× coverage), except for strain RM11, which corresponded to version 1 (ftp://ftp.sanger.ac.uk/pub/dmc/yeast/SGRP2/assembly/). In addition, sequences from wine strain EC1118 [24] were retrieved from GenBank database. Finally, *S. kudriavzevii* ZP591 and IFO 1802 sequences were downloaded from the *Saccharomyces sensu stricto* database (www.SaccharomycesSensuStricto.org).

### PCR Amplification and Sequencing

DNA was extracted following the procedure described by Querol *et al.* [25]. Genes *BRE5, CAT8, CYC3, CYR1*, *EGT2* and *GAL4* were amplified by PCR, using primers CAT8_3, CYR1_5, MET6_5, MET6_3, MET6_3 K from González *et al.* [16] and newly designed primers (Table S5), obtained from the comparison among sequences from strains *S. cerevisiae* S288C and *S. kudriavzevii* IFO 1802 and ZP591.

Most primers were species-specific with the exception of those for genes *CAT8*, *EGT2* and *GAL4*. The analysis of these genes required a previous step of cloning, performed by using a TOPO XL PCR Cloning Kit (Invitrogen). To detect the *S. cerevisiae* alleles in clones, a screening was carried out by colony-PCR with the corresponding primers, and a subsequent digestion of the PCR fragments following the procedure described in González *et al.* [16].

PCR amplifications were performed by using conditions described in González *et al.* [16] in a G-Storm Thermocycler (G-Storm Ltd, UK). Amplification products were cleaned with a High Pure PCR Product Purification Kit (Roche Diagnostics, Mannheim, Germany) and both strands of the DNA were directly sequenced using the BigDyeTM Terminator V3.0 Cycle Sequencing Kit (Applied Biosystems, Warrington, UK), following the manufacturer’s instructions in an Applied Biosystems automatic DNA sequencer Model ABI 3730l (Applied Biosystems). Sequences were edited and assembled with Staden Package v1.5 [26] to be deposited in GenBank under accession numbers JN709116 to JN709440.

### Haplotype and Haplogroup Classification

Gene sequences were aligned in MEGA 5 [27]. Haplotype classification was done in DnaSP v5 [28] using the previous haplotype number classification given by Arias [8]. New haplotypes were classified with consecutive Arabic numbers following the previous enumeration [8]. Median joining (MJ) networks [29] for *BRE5*, *CAT8*, *EGT2, GAL4* were constructed using Network 4.5 (http://www.fluxus-engineering.com/).

### Phylogenetic Analysis and Supernetworks

The neighbor-joining (NJ) and maximum-parsimony (MP) methods of phylogenetic reconstruction were applied to *BRE5*, *CAT8, CYC3, CYR1, EGT2, GAL4* and *MET6* separate sequence alignments of *S. cerevisiae* and *S. kudriavzevii* alleles from hybrid and reference strains described in Table S2. NJ trees were obtained with nucleotide distances corrected using the Maximum Composite Likelihood method. MP trees were obtained using the Close-Neighbor-Interchange algorithm in which the initial trees were obtained with the random addition of sequences (10 replicates). In all cases, a bootstrap analysis based on 2,000 pseudo-replicates was performed. For each gene, two NJ and MP phylogenetic trees were obtained, a tree based on *S. cerevisiae* alleles and another based on *S. kudriavzevii* alleles. Phylogenetic analyses were performed with MEGA 5 [27].

Two nexus files, with the collection of phylogenetic trees for *S. cerevisiae* and *S. kudriavzevii,* were created as an input of SPLITSTREE 4 package [30]. Two outputs corresponding to *S. cerevisiae* and *S. kudriavzevii* consensus super split networks (Supernetworks) were obtained, analyzing about 3.4 kb. For *S. cerevisiae* nexus file we reduced the number of splits setting maximum dimension parameter to 1, removing those splits in the network that are less supported. For the *S. kudriavzevii* nexus file we reduced the number of splits to simplify the final Supernetwork. For this simplification we applied the filtered Z-Closure method (filtering  = 2). A filter of 2 takes into account those splits that are compatible in at least 2 input trees in the nexus file. The result is a network that summarizes the relationships found in at least two trees simplifying the network [31].

### Array Competitive Genomic Hybridization (aCGH) and Flow Cytometry

Array competitive genomic hybridization (aCGH) experiments, scanning and data normalization were performed for IF6 and MR25 strains as previously described in Peris *et al.* [32]. A double-spotted array containing 6,240 ORFs of *S. cerevisiae* plus control spots totaling 6.4 K (Microarray Centre, University Health Network, Toronto, Canada) was used in aCGH assays. Raw and normalized microarray data are available in ArrayExpress [33], under accession number E-MEXP-3375.

Caryoscopes were obtained using ChARM v.1.1 [34]. Genome composition of IF6 and MR25 was inferred by combining aCGH (present study) and previous PCR-RFLPs data [17]. aCGH was performed following the procedure described in Peris *et al.* [32].

The approximate locations of the recombination points in the mosaic chromosomes were determined from the up and down jump locations in the ORFs mapping by microarray analysis of the hybrid yeast genomes. Collinearity between *S. kudriavzevii* and *S. cerevisiae* genomes [35], [36] allowed us to deduce S. *kudriavzevii* gene content in the hybrid genomes.

The list of *S. kudriavzevii* genes, excluding those with unknown function, retained in the hybrid genomes of IF6 and MR25 were independently analyzed using YeastMine in SGD database (http://yeastmine.yeastgenome.org:8080/yeastmine/begin.do) to obtain those Gene Ontology terms enriched in them. GO terms enrichment with p-values <0.05 were shown, after computing the Holm-Bonferroni for multiple hypothesis test correction. Significant GO terms were sorted according with their corresponding GO category.

The DNA content (C-value) of IF6 and MR25 was assessed by flow cytometry using a Beckman Coulter FC 500 (Beckman Coulter, USA) following the methodology described in Peris *et al.* [32]. Ploidy level was scored on the basis of the fluorescence intensity compared with the haploid *S. cerevisiae* S288c and diploid *S. cerevisiae* FY1679 reference strains.

### Maximum Parsimony Tree of Chromosomal Rearrangements

A list of minimal number of chromosomal rearrangements, chromosomal losses and restriction site changes for IF6 and MR25 strains obtained in this work as well as data obtained from Belloch *et al.* [37] and Peris *et al.* [17], [32] were included in the maximum parsimony analysis. A binary matrix was constructed to codify each particular event and these data were used to generate parsimony trees using MIX program from Phylip 3.66 package [38]. For this analysis, both chromosomal rearrangements and chromosomal gain/losses were considered as irreversible events (Camin-Sokal criterion), but data obtained from PCR-RFLP or sequence analyses were considered reversible events (Wagner criterion). The consensus tree was obtained by using the majority rule in the Consense program.

This binary matrix was also used to reconstruct a Median Joining Network, using Networks 4.5 (http://www.fluxus-engineering.com/), and a NeighborNet Phylonetwork, using SPLITSTREE 4 package [30].

## Results

### Phylogenetic Analysis of *S. cerevisiae* Genes from Hybrids

Phylogenetic relationships between *S. cerevisiae* x *S. kudriavzevii* natural hybrids obtained from several origins and a set of pure strains of the two parental species were analyzed to decipher possible common origins of these hybrids.

Nucleotide sequence data for both *S. cerevisiae* and *S. kudriavzevii* alleles of seven nuclear genes (*BRE5*, *CAT8*, *CYC3, CYR1, EGT2*, *GAL4,* and *MET6*) were obtained from a total of 24 natural *S. cerevisiae* x *S. kudriavzevii* and 2 *S. cerevisiae* x *S. kudriavzevii* x *S. uvarum* hybrid strains from several origins (Table S1). In a first phylogenetic analysis, we compared the *S. cerevisiae* sequences obtained for genes *BRE5*, *CAT8*, *EGT2* and *GAL4* from hybrids and from a representative selection, at the genotypic level, of 65 wine and 19 non-wine *S. cerevisiae* strains previously analyzed in our laboratory (Table S3 and Table S4). These genes were selected because they had shown high variability among *S. cerevisiae* strains from different origins [8]. Additionally, sequences from eight *S. cerevisiae* strains, five representative of the different “pure” lineages proposed by Liti *et al.* [9] and those from the completely sequenced genome of wine strain EC1118 [24] were also included in this study (Table S4).

**Figure 1:**
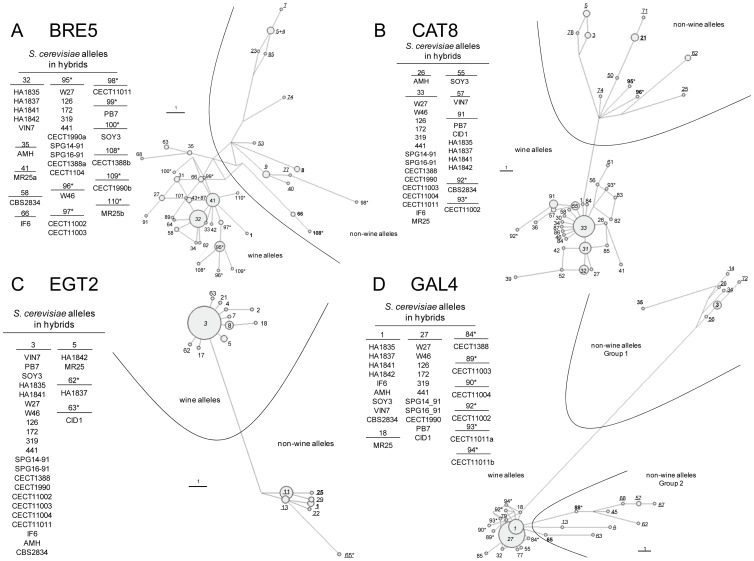
Median Joining (MJ) networks obtained for genes *BRE5* (A), *CAT8* (B), *EGT2* (c) and *GAL4* (D) from hybrid strains and representative wine and non-wine allele sequences according to Arias [**8**]. Strains representative of each different origin according to Liti et al. [9] and the alleles from wine strain EC1118 [24] were also included. Asterisks indicate new alleles not reported by Arias [8]. Numbers in italics indicate those alleles exhibited by wine strains from Liti *et al.* [9] and Novo *et al.* [24]. Numbers in bold indicate alleles present in non-wine strains from Liti *et al.* [9]. Underlined numbers correspond to alleles classified as “non-wine” in Arias [8].

Median-Joining networks (Figure 1) for all genes, except *GAL4*, showed two clearly differentiated groups of alleles or haplogroups. One haplogroup comprises those alleles present only in non-wine strains (so called non-wine alleles) and the second haplogroup includes alleles present in both wine and non-wine strains; however they are the only alleles exhibited by wine strains, and hence, they were called wine alleles. These wine alleles, when present in non-wine strains, are mainly found in heterozygosis with non-wine alleles. *GAL4* is the exception because non-wine alleles were clustered into two haplogroups. The first group is characterized by the presence of a common deleted region of 15 bp, and the second comprises different lineages and appears to be closer to the wine alleles than to haplogroup 1 (Figure 1D).

Fourteen *BRE5* alleles were present in hybrids (Figures 1 and S1), six are haplotypes already described in wine strains (32, 35, 41, 58, 66 and 95) and the other 8 were new alleles (96, 97, 98, 99, 100, 108, 109 and 110). MR25, CECT 1388 and CECT 1990 are heterozygous for this gene, exhibiting two wine *S. cerevisiae* alleles differing in one single nucleotide substitution (Figure S1). In the case of *CAT8*, 5 alleles from hybrids were present in wine yeasts (26, 33, 55, 57 and 91) and 2 were new (92 and 93). For *EGT2*, 2 alleles correspond to very common alleles in wine strains (3 and 5) and two were new (62 and 63), and finally *GAL4* showed a higher diversity in hybrids with 3 already known alleles (1, 18 and 27) and 6 new (84, 89, 90, 92, 93 and 94). These new alleles, found in hybrids for the first time, are indicated with asterisks in Figure 1. In general, alleles present in hybrids show few nucleotide differences (Figure S1) and are grouped together within the wine allele group for the four genes under analysis, with the exception of the *BRE5* new allele 98 from the brewing strain CECT11011 which is located within the non-wine haplogroup, probably due to the presence of 2 convergent nucleotide substitutions.

Strains DBVPG6044, Y12, YPS128 and UWOPS03-461.4 were selected as representative strains of the West African, Sake, North American and Malaysian pure populations of *S. cerevisiae*, respectively, as defined by Liti *et al.* [9]. Sequences from these strains (indicated in bold in Figure 1) always clustered within the non-wine group for the four genes analyzed. To the contrary, L1528, EC1118 and RM11, three wine strains representative of the pure Wine/European genotypic lineage defined by Liti *et al.* [9], always appear within the wine allele group (alleles indicated in italics in Figure 1). The laboratory strain S288c clustered within the wine (for *BRE5, CAT8* and *GAL4*) or non-wine groups (for *EGT2*) in accordance with its mosaic nature according to Liti *et al.* [9].

Because most *S. cerevisiae* alleles from hybrids are included within the wine allele group, the possible geographical origin of the hybrids was evaluated by analyzing the presence of these hybrid alleles in a set of 142 wine strains isolated from 8 different geographical areas, previously studied by Arias [8]. Table 1 shows the frequency of wine strains from each particular country sharing haplotypes with hybrids. The new alleles detected only in hybrids were not included in this analysis. As a general rule, the most frequent alleles in hybrids also corresponded to the most frequent alleles present in wine strains from several winemaking countries. For this reason, it is difficult to identify a specific geographic origin where hybridization processes may have occurred according to these comparisons (Table 1). Alleles 58 and 18 for *BRE5* and *GAL4* respectively were not found among the *S. cerevisiae* wine strains analyzed (Table 1), but they were detected in some non-wine strains (Table S4 and ref. [8]). However, these two alleles clustered within the wine allele groups (Figure 1 A and D).

**Table 1:** Frequency of wine strains isolated from different countries showing the same alleles found in hybrids.

Country	Total number of strains	Frequency (%) of each allele^a^														
		*BRE5*					*CAT8*				*EGT2*		*GAL4*			
		32*	35	41	58	66	26	33*	55	57	3*	5	1	18	27*	84
Argentina	37	49*	–	41	–	–	–	60*	3	–	81*	3	6	–	81*	3
Austria	30	30*	10	20	–	–	10	40*	–	–	60*	–	10	–	70*	–
Chile	23	23	–	41*	–	4	–	37*	4	–	81*	–	7	–	63*	–
France	13	23	–	46*	–	8	–	38*	23	–	92*	–	38	–	62*	–
Slovenia	5	20	–	–	–	–	–	20	–	–	80*	–	–	–	80*	–
South Africa	15	33*	–	–	–	–	–	33*	13	–	87*	–	33*	–	33*	–
Spain	14	21*	–	14	–	7	–	43*	14	21	93*	–	36	–	57*	–
Switzerland	5	40*	–	40*	–	–	20	–	–	–	80*	–	40*	–	40*	–

* The most frequent haplotype.

a Only those alleles present in more than one strain were included.

To identify how many putative *S. cerevisiae* parental strains were potentially involved in the origin of *S. cerevisiae* x *S. kudriavzevii* hybrids, we increased the number of genes analyzed in a second phylogenetic analysis. For this new analysis we included sequence data previously reported by Liti *et al.* [9] and Novo *et al.* [24] for comparative purposes.

Initial phylogenetic analyses on yeast were based on single gene sequences [39], but several times they failed to establish the overall history of these organisms. As an improvement, multigene sequence approaches using a concatenation of genes were proposed to construct the phylogenetic tree [40], [41]; however, they would represent an oversimplified version of the genetic history [30]. As an alternative, the construction of consensus trees has also been proposed, but this method can be only used when each gene tree has the same taxa representation [42]. In this work, because some hybrid strains have lost some particular *S. kudriavzevii* genes, both concatenated or consensus trees would oversimplify the results. Recently, a Z-closure method has been proposed to overcome this kind of problem [30], [31], [43], [44]. With this methodology, several gene trees with different taxa representation can be used as input files and a supernetwork with the complete set of taxa is obtained as [43]. However, one of the limitations of the Supernetwork analysis is the absence of statistical support, for this reason we interpreted our results according to a complementary phylogenetic analysis of the individual genes based on both Maximum Parsimony and Neighbor Joining. Both methods gave very similar or identical phylogenetic reconstructions (Figures S2 and S3).

**Figure 2:**
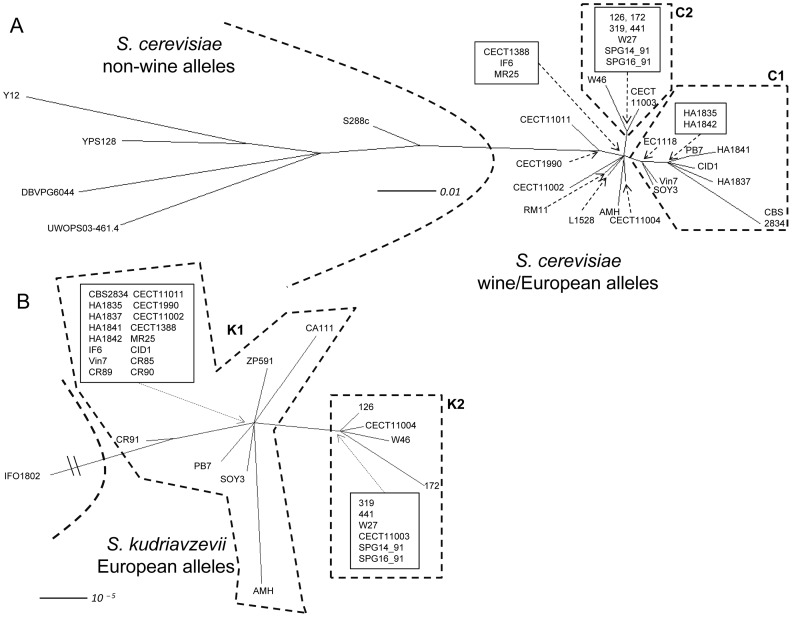
Supernetworks obtained using data from seven nuclear genes (*BRE5*, *CAT8*, *CYC3*, *CYR1*, *EGT2*, *GAL4* and *MET6*) for both *Saccharomyces cerevisiae* (A) and *Saccharomyces kudriavzevii* (B) alleles from hybrids, from reference *S. cerevisiae* [**9**], [**24**] and *S. kudriavzevii* strains. Scale bar represents the edge’s weights inferred using the tree size weighted means options, a measure similar to those from branches in a phylogenetic tree.

A supernetwork, containing the information of 7 *S. cerevisiae* nuclear genes (Figure 2A), showed two well defined groups of strains: a group comprising non-wine strains Y12, DBVPG6044, YPS128 and UWOPS03-461.4 and a group containing wine strains RM11, L1528, EC1118 and all hybrids (Figure 2A). The position of strain S288c in this supernetwork proved again ambiguous due to the mosaic nature of this strain.

According to this supernetwork analysis of *S. cerevisiae* gene sequences, hybrid strains appear clustered in two main subgroups (C1 and C2) and several independent lineages (Figure 2A). Subgroup C1 comprises Austrian (HA strains) and 3 other wine hybrids (PB7, SOY3 and Vin7), and the triple hybrids CID1 and CBS 2834, and subgroup C2 includes Swiss wine hybrids and Trappist beer strain CECT11003. The other hybrids appear in independent lineages (AMH, CECT 1990, 11002, 11004 and 11011) or in an ancestral position with respect to the two main subgroups (CECT1388, IF6 and MR25).

The supernetwork reconstruction method takes as input a set of complete or partial gene trees and produces a split network with the signals present in the gene trees, but it doesn’t allow to test the reliability of the the phylogenetic relationships. Therefore, bootstrap analyses for each individual gene Maximum-Parsimony and Neighbor-Joining trees were performed to contrast the confidence of these groupings (Supplemental Figures S2 and S3). Three of the seven genes (*CYC3*, *CYR1* and *EGT2*) showed low variability among hybrids and were useless to differentiate hybrid subgroups, although *EGT2,* together with *CAT8,* were the best genes to discriminate among wine and non-wine alleles. The remaining genes (*BRE5, CAT8, GAL4 and MET6*) differentiate subgroups of hybrids, but due to the low variability and the presence of putative convergent nucleotide substitutions, bootstrap values were low and did not support significantly many of these groupings.

In these individual gene trees (Figures S2 and S3), strains comprised in the supernetwork subgroup C1 (Swiss double hybrids and CECT 11003) are always included in the same cluster (Figure S2, alleles in blue), however, in the case of subgroup C2 (Figure S2, alleles in yellow), only Austrian hybrids, VIN7 and CBS2834 always appeared in the same cluster. The positions of the remaining strains change from one subgroup to the other, or to independent or intermediate lineages (Figure S2, alleles in green) depending on the gene (summarized in Table S6). As examples, wine hybrid SOY3 always appears within subgroup C1 group except for the *BRE*5 tree, where it is located in an intermediate position between wine and non-wine reference strains; W46 always appears within subgroup C2, except for *MET6* tree, in which it appears as part of subgroup C1; or CECT 1388 and 11002, which appear within subrgroup 1 in two gene trees but within subgroup 2 in the other 2.

### Phylogenetic Analysis of *S. kudriavzevii* Alleles from Hybrids

Another composite supernetwork was also obtained for the sequences of the *S. kudriavzevii* alleles present in hybrids. It is important to remark that *S. cerevisiae* x *S. kudriavzevii* hybrids are characterized by a trend to lose parts of the *S. kudriavzevii* subgenome [16], [17], [32], [37], and hence, some of the genes under analysis are absent in some strains. The most extreme case is strain AMH, which lost ∼72% of the S. kudriavzevii genome, and only maintains one of the seven genes under analysis (*CAT8*).

Homologous sequences from *S. kudriavzevii* pure strains isolated in Japan, Spain and Portugal were also included in the analysis (Table S1 and Table S2). This initial supernetwork was reconstructed without applying any filter (data not shown), however, a subsequent filtering was introduced to the analysis (see Methods section) to simplify the supernetwork analysis (Figure 2B). In this supernetwork, the European population represented by strains from Spain (CA111, CR85, CR89, CR90 and CR91) and Portugal (ZP591) forms a group far distant from the Japanese type strain IFO1802^T^ (Figure 2B). All *S. cerevisiae* x *S. kudriavzevii* hybrid strains were included within the European group. As in the case of the *S. cerevisiae* alleles, two main subgroups of hybrids are observed in this supernetwork. Subgroup K1 comprises most hybrids and occupies an ancestral position with respect to subgroup K2, including Swiss wine hybrids and Trappist beer hybrids CECT 11003 and 11004 (Figure 2B).

However, in the case of the *S. kudriavzevii* alleles, these groupings are better supported by the bootstrap analysis of Maximum-parsimony and Neighbor-Joining gene trees, even when nucleotide diversities are lower than in the case of *S. cerevisiae* alleles. In those trees based on variable genes *BRE5, CAT8, CYC3* and *CYR1*, Swiss wine hybrids and beer hybrids CECT 11003 and 11004 always appear within subgroup K2 (indicated in blue in Figure S2); and the wine hybrids from Austria (HA strains), VIN7 and SOY3 within subgroup K1 (indicated in yellow in Figure S2). In the case of hybrid IF6, this strain has lost two genes (*CAT8* and *CYC3*), but for the other genes it shares the same alleles than hybrids from subgroup K1 (Figure S1).

The positions of the remaining strains change from one subgroup to the other, or to independent positions (Figure S2, alleles in green) depending on the gene (summarized in Table S6). Thus, brewing hybrids CECT1388, 1990, and 11002, and the clinical isolate MR25 lost 1–2 genes (including the shared loss of *BRE5*). In the *CAT8* and *CYC3* trees, these strains appear within subgroup K1, but for *CYR1* they are included in a separate subgroup (indicated in green in Figure S2) due to the presence of allele 7, which differs from subgroup K1 allele 8 in a nucleotide substitution (Figure S1). Hybrid CECT11011 shares with the previous strains the *CYR1* allele 7 and their inclusion within subgroup K1 in the *CAT8* and *CYC3* trees, but within subgroup K2 in the *BRE5* tree, because maintains an allele identical to that from subgroup K2 strains. A similar situation is observed for triple hybrids CBS2834 and CID1, they appear within subgroup K2 in the *BRE5* tree but within subgroup K1 in the other gene trees, including *CYR1*. Finally, the Spanish wine hybrid PB7 appears within subgroup K1 in two gene trees (*CYC3* and *CYR1*), within subgroup K2 in other two (*BRE5* and *CAT8*), and it exhibits a different allele for *EGT2.*

### Genotypes of the Putative Parents of Hybrids Based on the Sequence Analysis of Seven Nuclear Genes

We tried to infer how many *S. cerevisiae* and *S. kudriavzevii* parents may have been involved in the generation of hybrids according to the phylogenetic analyses of the seven gene sequences. According to these sequences, the 24 double and 2 triple hybrids exhibit 20 different *S. cerevisiae* genotypes (allelic combinations) and 11 different *S. kudriavzevii* genotypes (Figure S1). These *S. cerevisiae* and *S. kudriavzevii* genotypes are found in 22 different combinations in hybrids. However, this does not mean that 22 different hybridization events occurred because hybrids are evolving after their origins. As seen before, the phylogenetic analysis of the sequences discriminate groups of alleles with putative common origins from an ancestral parental strain. In fact, the presence of rare alleles differing in few unique nucleotide substitutions (singletons) from the most common alleles in hybrids supports that these changes occurred after the hybridization process.

By considering the phylogenetic relationships among alleles and their combinations in hybrids (summarized in Table S6), we could infer 6 *S. cerevisiae* and 6 *S. kudriavzevii* putative ancestral genotypes (parental strains) that are arranged in 10 hybrid combinations (possible hybridization events). The first main hybrid combination is present in 6 wine hybrids, four from Austrian (HA strains), one from South Africa (VIN7, likely of European origin) and another from Croatia (SOY3). This SOY3 strain shares identical or closely related *S. cerevisiae* and *S. kudriavzevii* alleles with the other strains of this group for all genes except *BRE5*, which shows 4 nucleotide differences. This allele appears in the *BRE5* gene as closer to alleles from other hybrids (Figure 2S). These similarities could be explained by convergent substitutions, but we cannot rule out the possibility that the parental strain were originally heterozygous for *BRE5* and suffered a subsequent differential loss of heterozygosity in each derived hybrid lineage.

The second main combination is found in the 8 wine double hybrids from Switzerland and the Trappist beer hybrids CECT11003 and 11004 from Belgium. In this group, a slight discrepancy is also observed in strain CECT11004. This strain exhibits a *MET6* allele (allele 1) different to that present in other strains of this group (allele 2), but identical to that exhibited by strains from other groups (Figure S1). However, these *MET6* alleles 1 and 2 differ in one single synonymous substitution and a simple convergent change may explain this difference. An alternative explanation would be to consider allele *MET6*-1 as the ancestral one present in the *S. cerevisiae* parent of this group of hybrids later originating the derived allele *MET6*-2 shared by the Swiss and CECT11003 hybrids.

In the remaining hybrid combinations, both *S. cerevisiae* and *S. kudriavzevii* genotypes basically correspond to different arrangements of the alleles present in the first and second hybrid combinations described before. One explanation is that these recombining genotypes, generated by sexual mating at the within species level, were already present in the *S. cerevisiae* and *S. kudriavzevii* population before the hybridization events occurred. In this case, a minimum of 10 hybridization events would be necessary to explain the origin of these hybrids. However, another compatible explanation is that some hybrids may have originated by rare mating between diploid heterozygous cells, and a subsequent segregation of alleles due to chromosome loss (most hybrids are triploid [21], [32]), or random loss of heterozygosity due to recombination and/or gene conversion would generate the different mosaic hybrids. In this case, the number of hybridization events would be smaller than ten. This could be the case of brewing strains CECT1388, 1990, 11002, 11011 and the clinical isolate MR25. These strains exhibit similar *S. kudriavzevii* genotypes (including the specific allele *CYR1*-7), but different *S. cerevisiae* allele combinations, including wine and non-wine alleles (*CYR1*-2 and -4 in strains CECT1990 and 11011).

### The Genome Constitution of Non-fermentative Hybrids IF6 and MR25

In previous studies, we analyzed the genetic diversity of *S. cerevisiae x S. kudriavzevii* hybrids by RFLP analysis of 35 nuclear genes [16], [17] combined with array comparative genome hybridization (aCGH) [32], [37]. These analyses provided us information on the genome rearrangements occurred in the hybrids after their origins. Most of these rearrangements are non-reversible events that can complement the information obtained with the phylogenetic analysis of gene sequences to unveil the origin and evolution of these *S. cerevisiae* x *S. kudriavzevii* hybrids.

**Figure 3:**
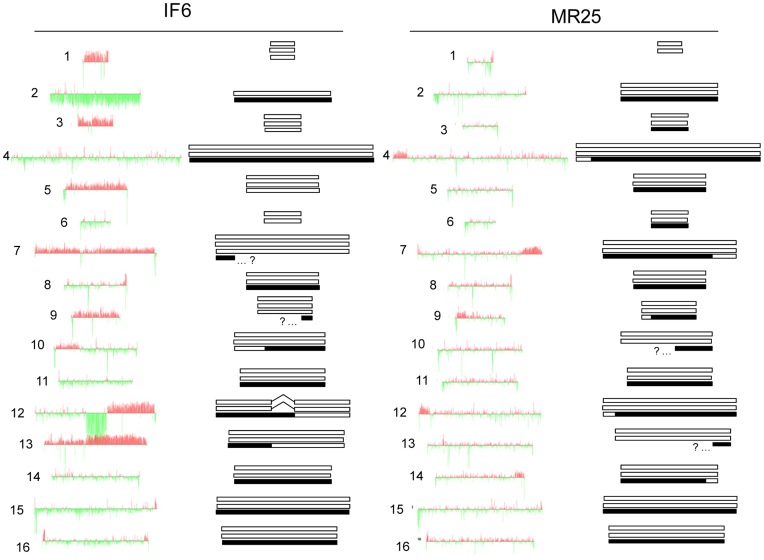
Genome composition of hybrid strains IF6 and MR25 obtained by combining aCGH (this work) and PCR-RFLP [**17**] analyses. Red and green signals correspond to the hybrid strain and the reference strain (S288c), respectively. White and black bars are used to represent *S. cerevisiae* and *S. kudriavzevii* fractions, respectively. Chromosomes showing black and white sections correspond to chimerical chromosomes. As an example, chromosome XIV in MR25 displayed a double RFLP pattern for *EGT2*, corresponding to the *S. cerevisiae* and *S. kudriavzevii* alleles, and one single pattern for *BRE5*, matching the *S. cerevisiae* allele restriction pattern [17]. The chimerical nature of this chromosome is confirmed by the caryoscope diagram where two different log_2_ ratios are observed, indicating a different *S. cerevisiae* chromosome content. By combining both sources of information, we can deduce that most chromosome XIV corresponds to two copies of *S. cerevisiae* (according to the *EGT2* RFLP pattern and aCGH data) and one of *S. kudriavzevii* (according to *EGT2* RFLP pattern), but chromosome XIV right end corresponds to three copies of *S. cerevisiae* (according to *BRE5* RFLP pattern and aCGH data). The recombination site in the chimerical chromosome can be located according to the log_2_ ratio jump observed in the caryoscope diagram.

However, the genome constitutions of hybrids IF6 and MR25 were not characterized in our previous studies, and therefore, they were subjected to aCGH and flow cytometry analyses to assess their genome compositions. Our results indicated that DNA content of IF6 and MR25 were 3.25 and 2.92 times that of the reference haploid strain S288c, respectively. These DNA content values, together with the aCGH analysis and PCR-RFLP data for 35 nuclear genes previously reported [17], allowed us to detect the presence of three chimerical chromosomes in hybrid IF6 (chr. X, XII and XIII) and five in MR25 (chr. IV, VII, IX, XII and XIV) (Figure 3). The hypothetical recombination points were mapped according to the *Saccharomyces* genome described in the SGD database (http://db.yeastgenome.org) using a window size of 15–20 Kb (four genes in the left and right of the most plausible recombination point). These recombination points were located in sequences corresponding to Ty LTRs, ARS and tRNAs (Table S7). RFLP analysis of genes located at the end of chromosomes [17] confirmed the presence of *S. kudriavzevii* segments in chromosomes VII and IX from IF6, and chromosomes X and XIII from MR25, however, their putative chimerical nature could not be detected by the aCGH analysis (Figure 3).

Following the same methodology used in our previous study [32], we obtained a list of *S. cerevisiae* genes lost in both hybrids IF6 and MR25. Both IF6 and MR25 have depleted a similar number of genes classified as retrotransposons as well as genes belonging to the *ASP3*, *CUP1* and *ENA* clusters (Table S8). In particular, hybrid IF6, obtained from a dietary supplement, exhibited a deleted region (YLR155C-YLR256W) in its *S. cerevisiae* chromosome XII (Figure 3). This region is adjacent to the rDNA repeat region located between YLR154C and YLR155C, which is not included in the microarray platform. A PCR amplification of the 5.8S-ITS region and the subsequent restriction analysis [45], revealed the absence of *S. cerevisiae* rDNA genes in this region (data not shown).

With respect to their *S. kudriavzevii* subgenome, IF6 and MR25 hybrids lost ∼33% and ∼18% of the total *S. kudriavzevii* genes, respectively. Gene Ontology (GO) enrichment analysis applied to the common set of *S. kudriavzevii* genes maintained by the two hybrids, demonstrated a high frequency of stress response genes among those *S. kudriavzevii* genes conserved in both hybrids (Table S9). Some of the significant GO terms shared by MR25 and IF6 are “response to stimulus” with p-values <0.05. In the case of MR25 is also important to note the significant GO term “cellular lipid metabolic process” and “response to stress” (p-value <0.05).

### Analysis of the Number of Hybridization Events

Genome composition data obtained for the 26 *S. cerevisiae* x *S. kudriavzevii* hybrids from this study as well as from previous studies [32], [37] were used to reconstruct a parsimony tree based on the presence of chimerical chromosomes, on the absence of chromosomes from one or another parental strain and the presence of specific allelic variants. Using the information from this parsimony tree together with the putative genetic constitution of the hypothetical parental strains obtained from the phylogenetic analysis of nuclear gene sequences, as well as from *COX2* sequences also obtained in our previous studies [17], allowed us to reduced the number of hybridization events to a minimum of six for the *S. cerevisiae* x *S. kudriavzevii* hybrids under analysis, and two additional events for the origin of the *S. cerevisiae* x *S. kudriavzevii* x *S. uvarum* triple hybrids. The putative ploidies of the parental cells involved in hybridization were also estimated by analyzing the genomic constitution of the hybrids derived from each event.

**Figure 4:**
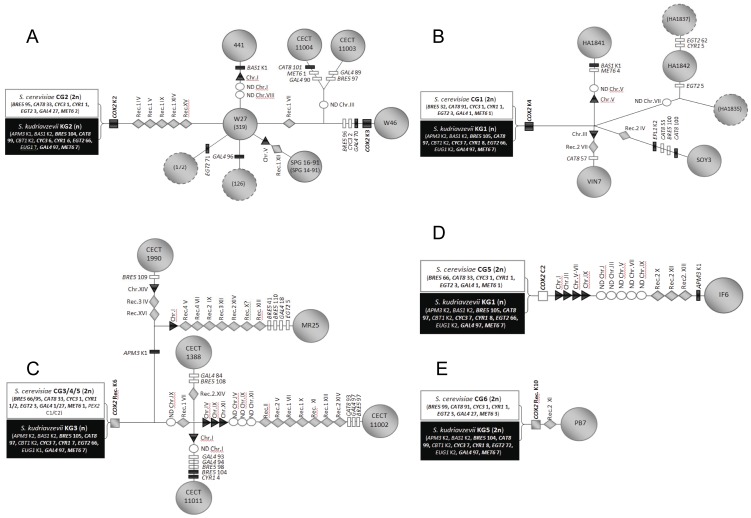
Possible multiple origins for hybrid strains based on Supernetworks, Polymorphic sites (Figure S1), Parsimony (Figure S2) and Neighbor-Joining (Figure S3) gene trees, PCR-RFLP data [**16**], [**17**], *COX2* sequence data [**17**] and maximum parsimony analysis of chromosome rearrangements [**32**]. Five out of six hybridization events are depicted in this figure, AMH and tripe hybrid origins have not been depicted due because they involved secondary hybridization events, in the case of AMH with another *S. cerevisiae* strain. The putative genetic backgrounds of the parental strains are indicated by squares on the left of each network. Symbols: triangles correspond to chromosome loss; squares to mitochondrial *COX2* haplotypes; diamonds to chromosome recombination events; rectangles to mutations generating new allele variants; circles to chromosome non-disjunctions. Those depicted in white are referring to events occurring in the *S. cerevisiae* subgenome of hybrids; in black, in the *S. kudriavzevii* subgenome; and in grey, those events involving both subgenomes (recombination events).

Figure 4 shows five out of the six different origins for double hybrids proposed according to this study. AMH is not included due to its complex genome structure, because it is a tetraploid hybrid that lost most of the *S. kudriavzevii* subgenome [17], [32]. Independent origin for AMH is clearly supported by the different sets of data used in this analysis.

Wine hybrid strains from Switzerland (W27, SPG14–91, SPG16–91,126, 172, 319 and 441), and the Trappist brewing strains CECT11003 and CECT11004 share a common origin. Their nuclear genomes derive from a hybridization event between the hypothetical *S. cerevisiae* CG2 and *S. kudriavzevii* KG2 parents (Figure S1). They inherited their mtDNA type K2 from *S. kudriavzevii* [17]. Hybrid W46 was also included in this group although it exhibits a mitochondrial type K3 (Figure 4), derived from K2 by a single nucleotide difference [16].

The group of Austrian hybrids HA as well as wine hybrids VIN7 and SOY3 have also a common origin in a hybridization event involving hypothetical parents *S. cerevisiae* CG1 and *S. kudriavzevii* KG1 (Figures 2, 4 and S1), and sharing the mitochondrial type K4 from *S. kudriavzevii* [17].

A third group includes the brewing triploid hybrids CECT1388, CECT1990, CECT11002, CECT11011 and the clinical isolate MR25, sharing several genome rearrangements and restriction patterns as well as a recombinant mtDNA type K6 [17]. According to the seven gene sequence analysis, these strains seem to have independent hybridization origins from crosses between different *S. cerevisiae* parents (CG3, CG4 and CG5) but the same *S. kudriavzevii* strain KG3 characterized by an specific *CYR1* allele (Figure S1). These contradictory results may be explained by considering a heterozygous *S. cerevisiae* diploid cell containing wine and non-wine alleles as the parental strain, as mentioned above.

Wine strain PB7 from Leon, Spain, was included in the same subgroup than the Austrian wine strains according to the supernetwork analyses, due to network simplification (Figure 2). However, this strain likely originated in an independent hybridization event because it derives from different parents, the mosaic *S. cerevisiae* CG6 and *S. kudriavzevii* KG5 genotypes (Figure S1), exhibits a recombinant mtDNA K10 [17], and finally, it possesses a tetraploid genome [32].

In the case of IF6, although it shares the same *S. kudriavzevii* KG1 ancestor with Austrian hybrids, its *S. cerevisiae* parental strain is clearly different: a mosaic CG5 genotype closer to the *S. cerevisiae* parent of the brewing hybrids. The different hybrid combination of parental genotypes supports an independent origin for this strain.

## Discussion

By analyzing the sequences of four nuclear genes from a total of more than 250 *S. cerevisiae* yeast isolates from wine (Europe, South America and South Africa) and non-wine origins (wild, brewing, cider, sake and traditional beverage fermentations mainly from Latin America, but also from Africa and Asia), Arias [8] demonstrated the existence of two groups of alleles, those present only in strains isolated from non-wine sources, called non-wine alleles, and another group of alleles that, while they also appear in non-wine alleles, they are the only alleles present in wine strains (wine alleles). These wine alleles are much less frequent in non-wine strains, and they mainly appear in heterozygosis with non-wine alleles. Liti *et al.* [9] obtained the complete genome sequences of 37 *S. cerevisiae* strains from different sources of isolation and geographic origins. The phylogenetic analysis of nucleotide polymorphisms showed a complex *S. cerevisiae* population structure. Liti *et al.* [9] observed five genotypic lineages, called according to their origins or source of isolation as Malaysia, West Africa, sake, North America and ‘Wine/European’, which exhibited the same phylogenetic relationships across their entire genomes. The strains from these five lineages were considered as ‘clean’, pure strains, representative of diverged populations. The other strains evidenced variable phylogenetic relationships depending on the genome region analyzed, and were considered as ‘mosaics’ with a mixed genome architecture that could be due to human traffic in yeast strains and subsequent recombination between them. The analysis of the sequences of the same four gene regions used by Arias [8] indicated that alleles present in the four non-wine lineages fell within the group of non-wine alleles; alleles present in strains of the wine/European lineage were included within the ‘wine allele’ group, and the locations of the ‘mosaic’ alleles were variable depending on the gene. Because Liti *et al.* [9] sequenced derivative monosporic cultures, some of the ‘mosaic’ parental strains could be heterozygous for wine and non wine alleles for many genes, as observed by Arias [8]. High levels of heterozygosity for non-wine yeast were also observed by Fay and Benavides [7], and for ale strains by Dunn and Sherlock [46].

The accessibility to such a collection of sequences (including genome sequences) from *S. cerevisiae* strains from different sources of isolation and geographic origins was an excellent opportunity to decipher the nature of the *S. cerevisiae* parents involved in the origin of hybrids. This way, for all genes under analysis, *S. cerevisiae* alleles from hybrids were always clustered within the wine allele group, with the exception of the *BRE5* allele from the brewing strain CECT11011, which clustered in the non-wine group, and *CYR1* allele from the brewing CECT1388 and CECT11011, which clustered with non-wine strains from Liti *et al.* (2009) in the individual gene trees. Moreover, the phylogenetic supernetwork analysis of *S. cerevisiae* alleles from hybrids identified two main subgroups of *S. cerevisiae* parental strains, and due to its simplification it failed to detect mosaic *S. cerevisiae* genotypes. It followed that the *S. cerevisiae* CG2 parental strain was involved in the hybridization event that originated the complete group of wine Swiss hybrids and *S. cerevisiae* CG1 was involved in the origin of the Austrian wine hybrids, SOY3 and Vin7.

The aCGH analyses of hybrid genome composition (present study and [32]) showed the depletion or underrepresentation of certain *S. cerevisiae* genes (Ty retrotransposons and *ENA* and *ASP* gene families), which were proposed as genomic signatures for wine *S. cerevisiae* yeasts [47], [48], which is in agreement with the postulated wine origin of the *S. cerevisiae* parental strains involved in the generation of these hybrids. The maintenance of *S. kudriavzevii* genes related to stress response, in MR25 and IF6, and lipid metabolism, in MR25, also confirms the importance of *S. kudriavzevii* subgenome in cold stress resistance, postulated in previous studies [32].

In the case of IF6, aCGH and PCR confirmation of 5.8S-ITS regions support the loss of heterozygosity (LOH) of the rDNA region in chromosome XII, maintaining only the *S. kudriavzevii* sequences for this region. This region has been characterized, in plants and animals, to be under concerted evolution [49]–[51]. This has been also observed in a natural hybrid *S. pastorianus* (CBS 1538 strain), where the *S. cerevisiae* rDNA region of chromosome XII has been lost [52].

The wine origin of the *S. cerevisiae* parent of most *S. cerevisiae* x *S. kudriavzevii* hybrids has already been postulated in previous works based on genomic composition data inferred by aCGH and PCR-RFLP analysis [32], as well as by microsatellite analysis [21]. The use of a multilocus sequence analysis approach certainly confirms the wine origin of the *S. cerevisiae* strains involved in the generation of most *S. cerevisiae* x *S. kudriavzevii* hybrids.

The exceptions are the brewing hybrid CECT11011, in which a possible recombinant *BRE5* allele is present, and CECT1990 and CECT11011, which contain *CYR1* non-wine alleles, and hence, a heterozygous non-wine *S. cerevisiae* strain, with both wine and non-wine alleles, could be involved in their origin. Dunn and Sherlock [46] demonstrated that *S. pastorianus* hybrids, responsible of lager beer fermentations, very likely derived from a cross between a haploid *S. bayanus*-like strain, later identified as belonging to the new species *S. eubayanus* [53], and a diploid *S. cerevisiae* strain, related to ale brewing strains, which are characterized by a high heterozygosity. Arias [8] also included in his study several ale strains that showed as heterozygous, for wine and non-wine alleles. Therefore, the parental *S. cerevisiae* involved in the origin of brewing hybrids CECT1990 and CECT11011 could be an ale strain originally heterozygous for wine and non-wine alleles. Another brewing hybrid, strain CECT11002, appeared as related to the brewing hybrids and the clinical isolate, but it did not contain non-wine alleles for the genes under analysis; all these hybrids may also have been originated from a similar ale parental strain. Erny *et al.* [21] included in their microsatellite analysis a Chimay strain which clusters with the *S. cerevisiae* brewing strains. We do not know whether their Chimay strain and our CECT11002 (also from Chimay) is the same or not, but at least they should be related, which could corroborate the ‘ale’ origin of their *S. cerevisiae* parent. Genome sequencing of one of these strains will elucidate this hypothesis.

By using the population genetic information from Arias [8], we also tried to determine the exact geographic origin of the parental *S. cerevisiae* strains. We looked for particular *S. cerevisiae* strains from different wine regions possessing the combination of alleles present in the hypothetical parental S. *cerevisiae* strains. With the exception of one *CAT8* allele, genotype CG1 was present in strains from Chile, South Africa, Switzerland and Spain; and genotypes CG2 and CG3, with the exception of *BRE5*, were found in strains from Argentina, Chile, Italy, Japan, South Africa, Austria, France and Spain. Other genotypes, with slight differences were found in Argentina, Chile, South Africa, Austria, Slovenia, Switzerland, Italy, Japan, France and Spain. As strains from the new winemaking regions (South America and South Africa in this case) were introduced from Europe with vines and winemaking tools, the most probable geographic origin for hybridization, according to the *S. cerevisiae* hypothetical parental genotype, is Europe.

The European origin of hybrids is also supported by the phylogenetic analysis of *S. kudriavzevii* alleles. Alleles present in hybrids were detected among European *S. kudriavzevii* pure strains. Three of seven alleles of *S. kudriavzevii* KG1, were found in 3 *S. kudriavzevii* strains from Ciudad Real (Spain), Castellon (Spain) [23] and Portugal [22]. However, other genotypes have not been found among the few *S. kudriavzevii* pure strains available. Future surveys on the genetic variability of European populations of *S. kudriavzevii* may be of interest to decipher the geographic origin of hybridization, because this wild species has not been subjected to human traffic and it may preserve its original population structure in the same way than *S. paradoxus* [54], [55]. A recent study [21], complementary to the present one, on the possible origin of a different set of European *S. cerevisiae* x *S. kudriavzevii* hybrids from winemaking (only four Swiss hybrids and VIN7 are in common), carried out by means of microsatellite information, also confirmed the European origin of the putative parental strains of hybrids.

By combining the phylogenetic analysis of gene sequences with all the available information on genetic and genomic characterization of *S. cerevisiae* x *S. kudriavzevii* hybrids [16], [17], [21], [32], [37], a total of six potential hybridization events were determined. The first hybridization event involved a haploid *S. kudriavevii* parental KG2 with mtDNA K2 and a diploid *S. cerevisiae* parental CG2. This event originated all Swiss hybrids and the related Trappist brewing strains CECT11003 and 11004. This clearly independent origin for Swiss wine hybrids is in accordance with the microsatellite phylogenetic analysis of hybrids performed by Erny *et al.* [21].

A second hybridization event involving a haploid *S. kudriavzevii* KG1 with mtDNA type K4 (found in all hybrids from this group) and a diploid *S. cerevisiae* CG1 originated a lineage of hybrids widely distributed in different wine regions such as Austrian hybrids, the Croatian strain SOY3, and the South African hybrid VIN7 of putative European origin according to Erny *et al.* [21]. These authors observed in their study that VIN7 is included in the same group as other Alsatian and German wine hybrids and bears a close relationship to Hungarian wine hybrids, confirming an European origin for VIN7. Therefore, this is a lineage of wine hybrids widely distributed from the Rhine valley (Alsace and Germany) to the Danube valley (Pannonian region: Austria, Croatia and Hungary).

A third hybridization event was involved in the origin of a lineage of brewing strains also widely distributed in ale breweries from England, Germany, Belgium (Chimay Trappist Abbey), New Zealand and the clinical isolate MR25. This hybridization event involved a haploid *S. kudriavzevii* parental close to K2, KG3 strain, and probably a heterozygous diploid *S. cerevisiae* parental. An “ale” *S. cerevisiae* strain heterozygous for wine and non-wine alleles could be involved in the origin of this group of hybrids.

Hybrid PB7 was probably originated from two diploid cells derived from mosaic strains *S. cerevisiae* CG6 and *S. kudriavzevii* KG5. Its tetraploidy [32] and the presence in this hybrid of a recombinant mtDNA [17] supports an independent hybridization event.

Independent origins are postulated for hybrids IF6 and AMH. In the case of AMH, its complex tetraploid genome [32], in which most of the *S. kudriavzevii* subgenome is lost [17], led us to suspect a possible scenario in which a diploid *S. cerevisiae* crossed with a haploid *S. kudriavzevii* strain and, after sporulation or a drastic *S. kudriavzevii* genome reduction, a diploid spore or an evolved derivative backcrossed with a diploid *S. cerevisiae.* IF6 was originated from a cross between a diploid *S. cerevisiae* CG5 mosaic genotype and a haploid *S. kudriavzevii* KG1, identical to the one involved in the origin of Austrian hybrids. Therefore, the possibility of a common origin with Austrian HA, VIN7, and SOY3 hybrids cannot completely ruled out if a heterozygous *S. cerevisiae* ancestor were involved in the hybridization event. However, this hypothesis not only requires the differential loss or segregation of alleles in the IF6 and Austrian lineages, but also the independent acquisition of the mitochondrial genome from the hybrid zygote, *S. cerevisiae* type C2 in IF6 and *S. kudriavzevii* type K4 in the Austrian lineage. This is possible in hybrid zygotes where three types of mitochondrial genomes may be present: two from each parental and a recombinant, generated after mitochondria fusion [56], but mitochondrial sorting occurs from the first budding formation [57], generating independent lineages that are difficult to distinguish from independent hybridizations in which parental relatives were involved.

Finally, triple hybrids *S. cerevisiae* x *S. kudriavzevii* x *S. uvarum* are not shown in Figure 4, also due to to their complex origins, in which a secondary hybridization was involved. However, the supernetwork analysis and gene trees information indicates that CBS2834 and CID1 were probably derived from the same (or similar) *S. kudriavzevii* parent (KG6) but different *S. cerevisiae* parental strains, the same than the Austrian strains (CG1) for CBS2834 and similar to PB7 (CG6) for CID1.

Finally, the origin of the triple hybrids CID1 and CBS2834 is not clear due to the additional occurrence of a secondary hybridization event either between a *S. cerevisiae* x *S. kudriavzevii* hybrid or derivative with a *S. uvarum* strain or between a *S. cerevisiae* x *S. uvarum* hybrid or derivative with a *S. kudriavzevii* strain. However, CID1 and CBS2834 were probably originated from independent hybridization events.

Most hybrids seem to have been generated by rare-mating events involving a diploid *S. cerevisiae* strain and a haploid strain of *S. kudriavzevii* generating different chimerical genomes with ploidy values close to 3 n. This is most clear for brewing strains (CECT1388, CECT1990, CECT11011 and MR25) where heterozygous genes could be observed. In PB7, which exhibited a ploidy value of 3.96, two diploid parents could be involved. Rare-mating has already been proposed as a mechanism for natural hybrid generation [58]. Additionally, artificial hybrids generated by rare mating are easily obtained in laboratory conditions [59].

Hybrid distribution and their physiological properties, together with the conclusions of recent studies on the population-genetic structure of *S. cerevisiae* [7], [9] as well as the phylogenetic analyses performed in the present study, can be used to speculate a possible scenario for the hybridization process. Grapevine [60] and barley [61] domestication mainly occurred in the Middle East, where the earliest archaeological evidence of winemaking [62] and brewing [63] have been discovered. From these areas of domestication, there was a gradual radiation to adjacent areas of the Mediterranean regions of Europe and Africa, following the spread of Phoenician, Greek and Carthaginian civilizations. Finally, the expansion of vine growing and winemaking to temperate regions of Oceanic and Continental climates of Europe, following the main trade fluvial routes, was performed under the influence of the Romans, who would take vine-growing to the limits of their empire, the Rhine and Danube Rivers. By the end of the Roman Empire, grape growing was common in most European locations. In the Middle Ages, viticulture and enology were improved and expanded by Christian monks.

Recent studies on the genetic diversity of *S. cerevisiae* populations [7]–[9], [64] show that wine strains constitute a genetically differentiated population that could have appeared during the process of adaptation to winemaking conditions, a process of fortuitous domestication of a *S. cerevisiae* wine strain. The microsatellite population analysis of *Saccharomyces* strains [65] also suggests that this population likely originated in the Near East and spread during the expansion of grapevine and winemaking.

About 2,000 years ago, wine *S. cerevisiae* yeasts were likely taken by the Romans, together with the vines and winemaking tools, to the Northern limit of grapevine distribution. There, *S. cerevisiae* wine strains, even nowadays, have problems when performing wine fermentations at the lower temperatures to which other *Saccharomyces* species are better adapted [66]. In these regions, cryotolerant species, such as *S. bayanus* var. *uvarum*, may outcompete *S. cerevisiae* [11], [67]–[69]. Under such circumstances, however, hybrids may have advantages over the parental species [19], [70], [71]. This is due to the acquisition of physiological properties from both parents, which provide a mechanism for selection of hybrids [10], [18], [72], [73]. In the case of *S. cerevisiae* x *S. kudriavzevii* hybrids, they acquired good alcohol and glucose tolerances and fast fermentation performances from *S. cerevisiae* [19], [74] and a better adaptation to low and intermediate temperatures from *S. kudriavzevii* [18], [19], [71].

These *S. cerevisiae* x *S. kudriavzevii* hybrids likely appeared several times, according to this study, and became frequent in some areas of the Northern limit of vine growing, but they could probably spread in Central Europe with the expansion of vine growing and winemaking practices that occurred during the Middle Ages [4]. Winemaking was preserved and improved during the Middle Ages by Christian monks. Benedictine abbeys were the main wine producers and traders, but the Cistercian reformation made possible the main revolution in winemaking improvements and vine growing extension [75].

From their original abbeys in Burgundy, Cistercians spread across Europe during the 11^th^ and 12^th^ centuries to establish more than 300 abbeys. During this expansion, the white monks spread the viticulture and enology practices to the Rhine and Danube valleys and the Pannonian basin of Central Europe [75]. They extended the Burgundian family of grape varieties, mainly Chardonnay and Pinots, as well as German varieties, and with them likely the hybrid yeasts responsible for wine fermentation.

In the regions where the main lineages of *S. cerevisiae* × *S. kudriavzevii* wine hybrids have been found, winemaking was introduced or improved on by Cistercian monks. In fact, the Cistercian order is given credit for planting in the French regions of Burgundy, Chablis, Loire, Rhone, Champagne (where the *S. cerevisiae* × *S. kudriavzevii* hybrid EPII, also called Epernay 2, was isolated [76]), Alsace (where many hybrids are also present and predominant [21]) and in several other wine regions in Central Europe. Some of these regions are: Rheingau Wine Region in Germany, where hybrid AMH (Assmannshausen) and those from Geisenheim [21] were isolated; Thermenregion, Austria, where HA hybrids, characterized in this study, were found as predominant in vineyards [15]; Slavonian Croatia, where SOY3 was isolated; and Hungary, where these hybrids have also been found [21].

## Supporting Information

Figure S1 **Polymorphic sites of the** ***S. cerevisiae*** **alleles (A) and** ***S. kudriavzevii*** **alleles (B) present in double and triple hybrids, as well as representative strains of the parental species.** Groups of hybrid alleles are colored according to their phylogenetic relationships based on the Maximum-Parsimony and Neighbor-Joining gene trees depicted in Figures S2 and S3, respectively. An asterisk indicates new allele not described in previous studies.
(DOCX)

Figure S2 **Maximum-Parsimony phylogenetic trees of 7 nuclear gene partial sequences of the** ***S. cerevisiae*** **(C) and** ***S. kudriavzevii*** **(K) subgenomes present in hybrids.** Sequences from pure strains representative of the parental species were also included in the analysis. *S. cerevisiae* reference strains isolated from wine and non-wine sources are indicated in red and blue, respectively. Numbers on the branches are nucleotide substitutions. Bootstrap values (in %) based on 2000 pseudo-replicates are given between parentheses. Groups of related hybrid alleles are highlighted in different colors, groups C1 and K1 in yellow, C2 and K2 in blue and C3 and K3 in green.
(PPTX)

Figure S3 ***S. cerevisiae*** **(C) and** ***S. kudriavzevii*** **(K) phylogenetic gene trees reconstructed using the Neighbor-joining method of the seven nuclear partial genes.** Scales are given in nucleotide substitutions per site, numbers on the nodes are bootstrap values (in %) based on 2000 pseudo-replicates. In the *S. cerevisiae* phylogenetic gene trees, representative wine and non-wine *S. cerevisiae* strains are colored in red and blue, respectively. In the case of *S. kudriavzevii* phylogenetic gene trees, Japanese and European *S. kudriavzevii* strains are indicated in red and blue, respectively. Groups of related hybrid alleles are indicated in different colors, groups C1 and K1 in yellow, C2 and K2 in blue and C3 and K3 in green.
(PPTX)

Table S1 **Geographic origins, source of isolation and genetic constitution of** ***Saccharomyces cerevisiae*** **x** ***Saccharomyces kudriavzevii*** **hybrids.**
(DOCX)

Table S2 **Geographic origins, source of isolation and genetic constitution of reference** ***Saccharomyces kudriavzevii*** **and** ***Saccharomyces cerevisiae*** **strains.** *S. cerevisiae* strains included in this Table correspond to representative strains belonging to the ‘pure’ lineages described by Liti *et al.* [9] based on their genome sequences, as well as to wine strain EC1118 [24].
(DOCX)

Table S3 **Geographic origin and genetic constitution of** ***Saccharomyces cerevisiae*** **wine strains isolated from different countries** [**8**] **.**
(DOCX)

Table S4 **Geographic origin, source of isolation and genetic constitution of** ***Saccharomyces cerevisiae*** **strains isolated from sources different from wine** [**8**] **.**
(DOCX)

Table S5 **PCR primers designed in the present study to amplify five nuclear gene regions.** PCR primers designed in the present study to amplify five nuclear gene regions. Those primers labeled with K and C are specific primers for *S. kudriavzevii* and *S. cerevisiae* alleles, respectively.
(DOCX)

Table S6 **Summarized results on the putative origin of hybrids based on the hybrid allele groups defined according to Maximum-Parsimony (Figure S2), and Neighbor-Joining (Figure S3) phylogenetic analyses of gene sequences.** Allele groups are highlighted in the same colors used to indicate allele groups in the Maximum-Parsimony and Neighbor-Joining gene trees depicted in Figures S2 and S3, respectively. Symbols: −, gene lost in the hybrid; 0, no group differentiation.
(DOCX)

Table S7 **List of chromosome rearrangements found in the dietary supplement IF6 and the clinical MR25 hybrid strains.**
(DOCX)

Table S8 **List of depleted** ***S. cerevisiae*** **genes detected in MR25 and IF6 hybrids.** The list shows genes found previously in hybrids characterized by Peris *et al.* [32] and compared against MR25 and IF6. Those genes depleted in MR25 and IF6 are indicated by X.
(XLSX)

Table S9 **Gene Ontology terms enriched in MR25 and IF6 using the** ***S. kudriavzevii*** **genes maintained in each genome.** Significant GO terms (p-value <0.05) were represented.
(XLSX)
