# Supplementary material for: Reconstruction of the Evolutionary History of Saccharomyces cerevisiae x S. kudriavzevii Hybrids Based on Multilocus Sequence Analysis
Source: PLoS One. 2012 Sep 25;7(9):e45527. doi: 10.1371/journal.pone.0045527 (PMC3458055; doi:10.1371/journal.pone.0045527)
Supplement: Figure S1 — Polymorphic sites of the S. cerevisiae alleles (A) and S. kudriavzevii alleles (B) present in double and triple hybrids, as well as representative strains of the parental species. Groups of hybrid alleles are colored according to their phylogenetic relationships based on the Maximum-Parsimony and Neighbor-Joining gene trees depicted in Figures S2 and S3, respectively. An asterisk indicates new allele not described in previous studies. (DOCX) [file pone.0045527.s001.docx]

**Figure S1. A**

|  |  |  |  | ***S. cerevisiae* alleles** | | | | | | | | | | |
| --- | --- | --- | --- | --- | --- | --- | --- | --- | --- | --- | --- | --- | --- | --- |
| **Strain** |  | **Genotype** |  | ***BRE5*** | **Allele** |  | ***CAT8*** | **Allele** |  | ***CYC3*** | **Allele** |  | ***CYR1*** | **Allele** |
| **EC1118** |  |  |  | **TAAGATCCGATCAAAA** |  |  | **CTATAAATTAACAGATCCCCTGCCCC** |  |  | **TGCCGA** | **1** |  | **TGAA** |  |
| **HA1835** |  | **CG1** |  | **.....G..........** | **32** |  | **.....................A....** | **91** |  | **......** | **1** |  | **....** | **1** |
| **HA1837** |  | **CG1** |  | **.....G..........** | **32** |  | **.....................A....** | **91** |  | **......** | **1** |  | **...C** | **5** |
| **HA1841** |  | **CG1** |  | **.....G..........** | **32** |  | **.....................A....** | **91** |  | **......** | **1** |  | **....** | **1** |
| **HA1842** |  | **CG1** |  | **.....G..........** | **32** |  | **.....................A....** | **91** |  | **......** | **1** |  | **....** | **1** |
| **VIN7** |  | **CG1** |  | **.....G..........** | **32** |  | **......G...................** | **57** |  | **......** | **1** |  | **....** | **1** |
| **SOY3** |  | **CG1** |  | **...C...G.....G..** | **100*** |  | **..........................** | **55** |  | **......** | **1** |  | **....** | **1** |
| **W27** |  | **CG2** |  | **CC...G.......G..** | **95*** |  | **...................T......** | **33** |  | **......** | **1** |  | **....** | **1** |
| **SPG14-91** |  | **CG2** |  | **CC...G.......G..** | **95*** |  | **...................T......** | **33** |  | **......** | **1** |  | **....** | **1** |
| **SPG16-91** |  | **CG2** |  | **CC...G.......G..** | **95*** |  | **...................T......** | **33** |  | **......** | **1** |  | **....** | **1** |
| **126** |  | **CG2** |  | **CC...G.......G..** | **95*** |  | **...................T......** | **33** |  | **......** | **1** |  | **....** | **1** |
| **172** |  | **CG2** |  | **CC...G.......G..** | **95*** |  | **...................T......** | **33** |  | **......** | **1** |  | **....** | **1** |
| **319** |  | **CG2** |  | **CC...G.......G..** | **95*** |  | **...................T......** | **33** |  | **......** | **1** |  | **....** | **1** |
| **441** |  | **CG2** |  | **CC...G.......G..** | **95*** |  | **...................T......** | **33** |  | **......** | **1** |  | **....** | **1** |
| **W46** |  | **CG2** |  | **CC...G..C....G..** | **96*** |  | **...................T......** | **33** |  | **G.....** | **2** |  | **....** | **1** |
| **CECT11003** |  | **CG2** |  | **C....G.......G..** | **97*** |  | **...................T......** | **33** |  | **......** | **1** |  | **....** | **1** |
| **CECT11004** |  | **CG2** |  | **CC...G.......G..** | **95*** |  | **...................T......** | **33** |  | **......** | **1** |  | **....** | **1** |
| **CECT1990** |  | **CG3** |  | **CC...K.......G..** | **95*,109*** |  | **...................T......** | **33** |  | **......** | **1** |  | **C...** | **2** |
| **CECT11011** |  | **CG3** |  | **CC...G....G.GGGG** | **98*** |  | **...................T......** | **33** |  | **......** | **1** |  | **CA..** | **4** |
| **CECT1388** |  | **CG4** |  | **CC...G....K..G..** | **95*,108*** |  | **...................T......** | **33** |  | **......** | **1** |  | **....** | **1** |
| **CECT11002** |  | **CG4** |  | **C....G.......G..** | **97*** |  | **.........T.........T..T...** | **93*** |  | **......** | **1** |  | **....** | **1** |
| **MR25** |  | **CG5** |  | **.....G....K..G..** | **41,110*** |  | **...................T......** | **33** |  | **......** | **1** |  | **....** | **1** |
| **IF6** |  | **CG5** |  | **.............G..** | **66** |  | **...................T......** | **33** |  | **......** | **1** |  | **....** | **1** |
| **AMH** |  | **CG5** |  | **.........T...GG.** | **35** |  | **...................T......** | **33** |  | **......** | **1** |  | **....** | **1** |
| **PB7** |  | **CG6** |  | **.....G.G.....G..** | **99*** |  | **.....................A....** | **91** |  | **......** | **1** |  | **....** | **1** |
| **CBS2834** |  | **CG1** |  | **.C...G..........** | **58** |  | **............T....T...A....** | **92*** |  | **......** | **1** |  | **....** | **1** |
| **CID1** |  | **CG7** |  |  | **‒** |  | **.....................A....** | **91** |  | **......** | **1** |  | **....** | **1** |
| **RM11** |  |  |  | **................** |  |  | **...................T....T.** |  |  | **......** |  |  | **....** |  |
| **L1528** |  |  |  | **.....G..........** |  |  | **...................T......** |  |  | **......** |  |  | **....** |  |
| **UWOPS03-461.4** |  |  |  | **......G......G--** |  |  | **T...G...C......C..TT.A.T..** |  |  | **.A....** |  |  | **C.T.** |  |
| **Y12** |  |  |  | **.............G--** |  |  | **TC...G.CC.G....C...T.A.T..** |  |  | **....C.** |  |  | **CA..** |  |
| **DBVPG6044** |  |  |  | **..G.G......T.GGG** |  |  | **T..C....C..T..TCT..TCA.T..** |  |  | **...T.G** |  |  | **C...** |  |
| **YPS128** |  |  |  | **.............G--** |  |  | **T.......C....A.C...T.A.T.T** |  |  | **..A...** |  |  | **C...** |  |

**Figure S1. A,** Cont.

|  |  |  |  | ***S. cerevisiae* alleles** | | | | | | | |
| --- | --- | --- | --- | --- | --- | --- | --- | --- | --- | --- | --- |
| **Strain** |  | **Genotype** |  | ***EGT2*** | **Allele** |  | ***GAL4*** | **Allele** |  | ***MET6*** | **Allele** |
| **EC1118** |  |  |  | **CAATTTATCCTGAA** |  |  | **TCGAGGACGCAAGGCCGT** |  |  | **GATCAT** |  |
| **HA1835** |  | **CG1** |  | **..............** | **3** |  | **..................** | **1** |  | **......** | **1** |
| **HA1837** |  | **CG1** |  | **.........A....** | **62*** |  | **..................** | **1** |  | **......** | **1** |
| **HA1841** |  | **CG1** |  | **..............** | **3** |  | **..................** | **1** |  | **.GA...** | **4** |
| **HA1842** |  | **CG1** |  | **T.............** | **5** |  | **..................** | **1** |  | **......** | **1** |
| **VIN7** |  | **CG1** |  | **..............** | **3** |  | **..................** | **1** |  | **......** | **1** |
| **SOY3** |  | **CG1** |  | **..............** | **3** |  | **..................** | **1** |  | **......** | **1** |
| **W27** |  | **CG2** |  | **..............** | **3** |  | **.T..AC............** | **27** |  | **...T..** | **2** |
| **SPG14-91** |  | **CG2** |  | **..............** | **3** |  | **.T..AC............** | **27** |  | **...T..** | **2** |
| **SPG16-91** |  | **CG2** |  | **..............** | **3** |  | **.T..AC............** | **27** |  | **...T..** | **2** |
| **126** |  | **CG2** |  | **..............** | **3** |  | **.T..AC............** | **27** |  | **...T..** | **2** |
| **172** |  | **CG2** |  | **..............** | **3** |  | **.T..AC............** | **27** |  | **...T..** | **2** |
| **319** |  | **CG2** |  | **..............** | **3** |  | **.T..AC............** | **27** |  | **...T..** | **2** |
| **441** |  | **CG2** |  | **..............** | **3** |  | **.T..AC............** | **27** |  | **...T..** | **2** |
| **W46** |  | **CG2** |  | **..............** | **3** |  | **.T..AC............** | **27** |  | **...T..** | **2** |
| **CECT11003** |  | **CG2** |  | **..............** | **3** |  | **....AC..........A.** | **89*** |  | **...T..** | **2** |
| **CECT11004** |  | **CG2** |  | **..............** | **3** |  | **....AC......A.....** | **90*** |  | **......** | **1** |
| **CECT1990** |  | **CG3** |  | **..............** | **3** |  | **.T..AC............** | **27** |  | **......** | **1** |
| **CECT11011** |  | **CG3** |  | **..............** | **3** |  | **....AS.........A..** | **93*,94*** |  | **......** | **1** |
| **CECT1388** |  | **CG4** |  | **..............** | **3** |  | **.T................** | **84** |  | **......** | **1** |
| **CECT11002** |  | **CG4** |  | **..............** | **3** |  | **...............G..** | **92*** |  | **......** | **1** |
| **MR25** |  | **CG5** |  | **T.............** | **5** |  | **.....C............** | **18** |  | **......** | **1** |
| **IF6** |  | **CG5** |  | **..............** | **3** |  | **..................** | **1** |  | **......** | **1** |
| **AMH** |  | **CG5** |  | **..............** | **3** |  | **..................** | **1** |  | **......** | **1** |
| **PB7** |  | **CG6** |  | **..............** | **3** |  | **.T..AC............** | **27** |  | **..A...** | **3** |
| **CBS2834** |  | **CG1** |  | **..............** | **3** |  | **..................** | **1** |  | **....TC** | **5** |
| **CID1** |  | **CG6** |  | **.............G** | **63*** |  | **.T..AC............** | **27** |  | **......** | **1** |
| **RM11** |  |  |  | **..............** |  |  | **.T..AC............** |  |  | **....** |  |
| **L1528** |  |  |  | **..............** |  |  | **.T..AC............** |  |  | **....** |  |
| **UWOPS03-461.4** |  |  |  | **.TGCC.G.T.CTT.** |  |  | **C..G..............** |  |  | **A...** |  |
| **Y12** |  |  |  | **.TGCCC.CT...T.** |  |  | **C..G...TAT.T.....C** |  |  | **....** |  |
| **DBVPG6044** |  |  |  | **.TGCCC..T.C.T.** |  |  | **..T..C.T.....C....** |  |  | **....** |  |
| **YPS128** |  |  |  | **TTGCCC..T.C.T.** |  |  | **......G...G...T..C** |  |  | **....** |  |

**Figure S1. B**.

|  |  |  |  | ***S. kudriavzevii* alleles** | | | | | | | | | | | | | | | | |
| --- | --- | --- | --- | --- | --- | --- | --- | --- | --- | --- | --- | --- | --- | --- | --- | --- | --- | --- | --- | --- |
| **Strain** |  | **Genotype** |  | ***BRE5*** | **Allele** |  | ***CAT8*** | **Allele** |  | ***CYC3*** | **Allele** |  | ***CYR1*** | **Allele** |  | ***EGT2*** | **Allele** |  | ***GAL4*** | **Allele** |
| **CR85** |  |  |  | **TTACC** |  |  | **ACGAGATAC** |  |  | **CT** |  |  | **TGT** |  |  | **AACTGC** |  |  | **GGA** |  |
| **HA1835** |  | **KG1** |  | **....T** | **105** |  | **.........** | **97** |  | **..** | **7** |  | **...** | **8** |  | **....A.** | **66** |  | **C..** | **97** |
| **HA1837** |  | **KG1** |  | **....T** | **105** |  | **.........** | **97** |  | **..** | **7** |  | **...** | **8** |  | **....A.** | **66** |  | **C..** | **97** |
| **HA1841** |  | **KG1** |  | **....T** | **105** |  | **.........** | **97** |  | **..** | **7** |  | **...** | **8** |  | **....A.** | **66** |  | **C..** | **97** |
| **HA1842** |  | **KG1** |  | **....T** | **105** |  | **.........** | **97** |  | **..** | **7** |  | **...** | **8** |  | **....A.** | **66** |  | **C..** | **97** |
| **VIN7** |  | **KG1** |  | **....T** | **105** |  | **.........** | **97** |  | **..** | **7** |  | **...** | **8** |  | **....A.** | **66** |  | **C..** | **97** |
| **SOY3** |  | **KG1** |  | **....T** | **105** |  | **.T......T** | **100** |  | **..** | **7** |  | **...** | **8** |  | **....A.** | **66** |  | **C..** | **97** |
| **IF6** |  | **KG1** |  | **....T** | **105** |  |  | **‒** |  |  | **‒** |  | **...** | **8** |  | **....A.** | **66** |  | **C..** | **97** |
| **W27** |  | **KG2** |  | **..G.T** | **104** |  | **..C.C....** | **99** |  | **T.** | **6** |  | **.AC** | **6** |  | **....A.** | **66** |  | **C..** | **97** |
| **SPG14-91** |  | **KG2** |  | **..G.T** | **104** |  | **..C.C....** | **99** |  | **T.** | **6** |  | **.AC** | **6** |  | **....A.** | **66** |  | **C..** | **97** |
| **SPG16-91** |  | **KG2** |  | **..G.T** | **104** |  | **..C.C....** | **99** |  | **T.** | **6** |  | **.AC** | **6** |  | **....A.** | **66** |  | **C..** | **97** |
| **126** |  | **KG2** |  | **..G.T** | **104** |  | **..C.C....** | **99** |  | **T.** | **6** |  | **.AC** | **6** |  | **....A.** | **66** |  | **CC.** | **96** |
| **172** |  | **KG2** |  | **..G.T** | **104** |  | **..C.C....** | **99** |  | **T.** | **6** |  | **.AC** | **6** |  | **..GGA.** | **71** |  | **C..** | **97** |
| **319** |  | **KG2** |  | **..G.T** | **104** |  | **..C.C....** | **99** |  | **T.** | **6** |  | **.AC** | **6** |  | **....A.** | **66** |  | **C..** | **97** |
| **441** |  | **KG2** |  | **..G.T** | **104** |  | **..C.C....** | **99** |  |  | **‒** |  | **.AC** | **6** |  | **....A.** | **66** |  | **C..** | **97** |
| **W46** |  | **KG2** |  | **..G.T** | **104** |  | **..C.C....** | **99** |  | **T.** | **6** |  | **.AC** | **6** |  | **.C..A.** | **70** |  | **C..** | **97** |
| **CECT11003** |  | **KG2** |  | **..G.T** | **104** |  | **..C.C....** | **99** |  | **T.** | **6** |  | **.AC** | **6** |  | **....A.** | **66** |  | **C..** | **97** |
| **CECT11004** |  | **KG2** |  | **..G.T** | **104** |  | **T.C.C....** | **101** |  | **T.** | **6** |  | **.AC** | **6** |  | **....A.** | **66** |  | **C..** | **97** |
| **CECT1990** |  | **KG3** |  |  | **‒** |  | **.........** | **97** |  | **..** | **7** |  | **C..** | **7** |  |  | **‒** |  | **C..** | **97** |
| **CECT11011** |  | **KG3** |  | **..G.T** | **104** |  | **.........** | **97** |  |  | **‒** |  | **C..** | **7** |  | **....A.** | **66** |  | **C..** | **97** |
| **CECT1388** |  | **KG3** |  |  | **‒** |  | **.........** | **97** |  | **..** | **7** |  | **C..** | **7** |  | **....A.** | **66** |  | **C..** | **97** |
| **CECT11002** |  | **KG3** |  |  | **‒** |  |  | **‒** |  | **..** | **7** |  | **C..** | **7** |  | **....A.** | **66** |  | **C..** | **97** |
| **MR25** |  | **KG3** |  |  | **‒** |  | **.........** | **97** |  |  | **‒** |  | **C..** | **7** |  | **....A.** | **66** |  | **C..** | **97** |
| **PB7** |  | **KG4** |  | **..G.T** | **104** |  | **..C.C....** | **99** |  | **..** | **7** |  | **...** | **8** |  | **T.....** | **72** |  | **C..** | **97** |
| **AMH** |  | **KG5** |  |  | **‒** |  | **......C..** | **98** |  |  | **‒** |  |  | **‒** |  |  | **‒** |  |  | **‒** |
| **CBS2834** |  | **KG6** |  | **..G.T** | **104** |  | **.........** | **97** |  |  | **‒** |  | **...** | **8** |  |  | **‒** |  | **C..** | **97** |
| **CID1** |  | **KG6** |  | **..G.T** | **104** |  | **.........** | **97** |  | **..** | **7** |  | **...** | **8** |  | **....A.** | **66** |  |  | **‒** |
| **CR89** |  |  |  | **.....** |  |  | **....CG...** |  |  | **..** |  |  | **.A.** |  |  | **....AA** |  |  | **...** |  |
| **CR90** |  |  |  | **.....** |  |  | **....CG...** |  |  | **..** |  |  | **.A.** |  |  | **....AA** |  |  | **C.G** |  |
| **CR91** |  |  |  | **....T** |  |  | **...GCG...** |  |  | **..** |  |  | **...** |  |  | **......** |  |  | **...** |  |
| **CA111** |  |  |  | **.G..T** |  |  | **....C..G.** |  |  | **.G** |  |  | **...** |  |  | **....AA** |  |  | **C..** |  |
| **ZP591** |  |  |  | **C..TT** |  |  | **....CG...** |  |  | **..** |  |  | **...** |  |  | **....A.** |  |  | **C.G** |  |

Groups of hybrid alleles are colored according to their phylogenetic relationships based on the Maximum-Parsimony and Neighbor-Joining gene trees depicted in Figures S2 and S3, respectively.
