# Supplementary material for: Reconstruction of the Evolutionary History of Saccharomyces cerevisiae x S. kudriavzevii Hybrids Based on Multilocus Sequence Analysis
Source: PLoS One. 2012 Sep 25;7(9):e45527. doi: 10.1371/journal.pone.0045527 (PMC3458055; doi:10.1371/journal.pone.0045527)
Supplement: Table S1 — Geographic origins, source of isolation and genetic constitution of Saccharomyces cerevisiae x Saccharomyces kudriavzevii hybrids. (DOCX) [file pone.0045527.s004.docx]

| **Table S1.** Geographic origins, source of isolation and genetic constitution of *Saccharomyces cerevisiae* x *Saccharomyces kudriavzevii* hybrids. | | | | | | | | | | | |  |
| --- | --- | --- | --- | --- | --- | --- | --- | --- | --- | --- | --- | --- |
| **Strain** | **Hybrid type^a^** | **Country** | **Isolation source** |  | **Alleles (*S. cerevisiae* / *S. kudriavzevii*)^b^** | | | | | | | |
|  |  |  |  |  | ***BRE5*** | ***CAT8*** | ***CYC3*** | ***CYR1*** | ***EGT2*** | ***GAL4*** | ***MET6*** | |
| HA 1835 | Sc/Sk | Austria | Wine |  | 32 / 105 | 91* / 97 | 1 / 7 | 1 / 8 | 3 / 66 | 1 / 97 | 1 / 7 | |
| HA 1837 | Sc/Sk | Austria | Wine |  | 32 / 105 | 91* / 97 | 1 / 7 | 5 / 8 | 62* / 66 | 1 / 97 | 1 / 7 | |
| HA 1841 | Sc/Sk | Austria | Wine |  | 32 / 105 | 91* / 97 | 1 / 7 | 1 / 8 | 3 / 66 | 1 / 97 | 4 / - | |
| HA 1842 | Sc/Sk | Austria | Wine |  | 32 / 105 | 91* / 97 | 1 / 7 | 1 / 8 | 5 / 66 | 1 / 97 | 1 / 7 | |
| VIN7 | Sc/Sk | South Africa | Wine |  | 32 / 105 | 57 / 97 | 1 / 7 | 1 / 8 | 3 / 66 | 1 / 97 | 1 / 7 | |
| SOY3 | Sc/Sk | Croatia | Wine |  | 100* / 105 | 55 / 100 | 1 / 7 | 1 / 8 | 3 / 66 | 1 / 97 | 1 / 7 | |
| W27 | Sc/Sk | Switzerland | Wine |  | 95* / 104 | 33 / 99 | 1 / 6 | 1 / 6 | 3 / 66 | 27 / 97 | 2 / 7 | |
| W46 | Sc/Sk | Switzerland | Wine |  | 96* / 104 | 33 / 99 | 2 / 6 | 1 / 6 | 3 / 70 | 27 / 97 | 2 / 7 | |
| SPG 14_91 | Sc/Sk | Switzerland | Wine |  | 95* / 104 | 33 / 99 | 1 / 6 | 1 / 6 | 3 / 66 | 27 / 97 | 2 / 7 | |
| SPG 16_91 | Sc/Sk | Switzerland | Wine |  | 95* / 104 | 33 / 99 | 1 / 6 | 1 / 6 | 3 / 66 | 27 / 97 | 2 / 7 | |
| 126 | Sc/Sk | Switzerland | Wine |  | 95* / 104 | 33 / 99 | 1 / 6 | 1 / 6 | 3 / 66 | 27 / 96 | 2 / 7 | |
| 172 | Sc/Sk | Switzerland | Wine |  | 95* / 104 | 33 / 99 | 1 / 6 | 1 / 6 | 3 / 71 | 27 / 97 | 2 / 7 | |
| 319 | Sc/Sk | Switzerland | Wine |  | 95* / 104 | 33 / 99 | 1 / 6 | 1 / 6 | 3 / 66 | 27 / 97 | 2 / 7 | |
| 441 | Sc/Sk | Switzerland | Wine |  | 95* / 104 | 33 / 99 | 1 / - | 1 / 6 | 3 / 66 | 27 / 97 | 2 / 7 | |
| AMH | Sc/Sk | Germany | Wine |  | 35 / - | 26 / 98 | 1 / - | 1 / - | 3 / - | 1 / - | 1 / - | |
| PB7 | Sc/Sk | Spain | Wine |  | 99* / 104 | 91* / 99 | 1 / 7 | 1 / 8 | 3 / 72 | 27 / 97 | 3 / 7 | |
| CECT 1388^†^ | Sc/Sk | England | Beer |  | 95* 108* / - | 33 / 97 | 1 / 7 | 1 / 7 | 3 / 66 | 84 / 97 | 1 / 7 | |
| CECT 1990^†^ | Sc/Sk | Germany | Beer |  | 95* 109* / - | 33 / 97 | 1 / 7 | 2 / 7 | 3 / - | 27 / 97 | 1 / 7 | |
| CECT 11002 | Sc/Sk | Belgium | Beer |  | 97* / - | 93* / - | 1 / 7 | 1 / 7 | 3 / 66 | 92* / 97 | 1 / 7 | |
| CECT 11003 | Sc/Sk | Belgium | Beer |  | 97* / 104 | 33 / 99 | 1 / 6 | 1 / 6 | 3 / 66 | 89* / 97 | 2 / 7 | |
| CECT 11004 | Sc/Sk | Belgium | Beer |  | 95* / 104 | 33 / 101 | 1 / 6 | 1 / 6 | 3 / 66 | 90* / 97 | 1 / 7 | |
| CECT 11011^†^ | Sc/Sk | New Zealand | Beer |  | 98* / 104 | 33 / 97 | 1 / - | 4 / 7 | 3 / 66 | 93* 94 / 97 | 1 / 7 | |
| MR25^†^ | Sc/Sk | Spain | Respiratory tract |  | 41 110* / - | 33 / 97 | 1 / - | 1 / 7 | 5 / 66 | 18 / 97 | 1 / 7 | |
| IF6 | Sc/Sk | Spain | Dietary complement |  | 66 / 105 | 33 / - | 1 / - | 1 / 8 | 3 / 66 | 1 / 97 | 1 / - | |
| CBS 2834 | Sc/Sk/Su | Switzerland | Wine |  | 58 / 104 | 92* / 97 | 1 / - | 1 / 8 | 3 / - | 1 / 97 | 5 / 7 | |
| CID1 | Sc/Sk/Su | France | Cider |  | - / 104 | 91* / 97 | 1 / 7 | 1 / 8 | 63* / 66 | 27 / nd | 1 / 7 | |
| a- Sc: *Saccharomyces cerevisiae*; Sk: *Saccharomyces kudriavevii*; Su: *Saccharomyces uvarum*.  b- Symbol “- “ indicates the absence of the *S. kudriavzevii* gene.  *New *S. cerevisiae* alleles for those 4 genes surveyed in a previous study [8].  ^†^ Heterozygous for some *S. cerevisiae* genes. | | | | | | | | | | | |  |
