# Supplementary material for: Reconstruction of the Evolutionary History of Saccharomyces cerevisiae x S. kudriavzevii Hybrids Based on Multilocus Sequence Analysis
Source: PLoS One. 2012 Sep 25;7(9):e45527. doi: 10.1371/journal.pone.0045527 (PMC3458055; doi:10.1371/journal.pone.0045527)
Supplement: Table S2 — Geographic origins, source of isolation and genetic constitution of reference Saccharomyces kudriavzevii and Saccharomyces cerevisiae strains. S. cerevisiae strains included in this Table correspond to representative strains belonging to the ‘pure’ lineages described by Liti et al. [9] based on their genome sequences, as well as to wine strain EC1118 [24]. (DOCX) [file pone.0045527.s005.docx]

| **Table S2.** Geographic origins, source of isolation and genetic constitution of reference *Saccharomyces kudriavzevii* and *Saccharomyces cerevisiae* strains. *S. cerevisiae* strains included in this Table correspond to representative strains belonging to the ‘pure’ lineages described by Liti *et al.* [9] based on their genome sequences, as well as to wine strain EC1118 [24] | | | | | | | | | | |
| --- | --- | --- | --- | --- | --- | --- | --- | --- | --- | --- |
| **Strain** | **Specie** | **Country** | **Isolation source** | **Alleles** | | | | | | |
|  |  |  |  | ***BRE5*** | ***CAT8*** | ***CYC3*** | ***CYR1*** | ***EGT2*** | ***GAL4*** | ***MET6*** |
| IFO 1802 | *S. kudriavzevii* | Japan | Decayed leaf | 103 | 105 | 8 | 10 | 72 | Ψ | 6 |
| ZP 591 | *S. kudriavzevii* | Portugal | *Quercus* sp. | 102 | 102 | 7 | 8 | 66 | 95 | 7 |
| CR 85 | *S. kudriavzevii* | Spain | *Quercus ilex* bark | 107 | 97 | 7 | 8 | 68 | 98 | 7 |
| CR 89 | *S. kudriavzevii* | Spain | *Quercus faginea* bark | 107 | 102 | 7 | 9 | 67 | 98 | 7 |
| CR 90 | *S. kudriavzevii* | Spain | *Q. faginea* bark | 107 | 102 | 7 | 9 | 67 | 95 | 7 |
| CR 91 | *S. kudriavzevii* | Spain | *Q. faginea* bark | 105 | 103 | 7 | 8 | 68 | 98 | 7 |
| CA 111 | *S. kudriavzevii* | Spain | *Q. ilex* bark | 106 | 104 | 9 | 8 | 67 | 97 | 7 |
| Y12 | *S. cerevisiae* | Japan | Sake | 66* | 21 | 4 | 4 | 11 | 35 | 1 |
| YPS128 | *S. cerevisiae* | USA | *Quercus* sp. | 66* | 96 | 4 | 2 | 1 | 88 | 1 |
| DBVPG6044 | *S. cerevisiae* | West Africa | Bili wine | 8 | 62 | 5 | 2 | 13 | 3 | 1 |
| UWOPS03 | *S. cerevisiae* | Malaysia | Nectar, Bertam palm | 108* | 95 | 3 | 3 | 65 | 65 | 8 |
| S288c | *S. cerevisiae* | USA | Lab Strain | 1 | 1 | 1 | 2 | 1 | 1 | 1 |
| L1528 | *S. cerevisiae* | Chile | Wine | 32 | 33 | 10 | 1 | 1 | 27 | 1 |
| RM11 | *S. cerevisiae* | USA | Vineyard | 32 | 31 | 1 | 1 | 3 | 27 | 1 |
| EC1118 | *S. cerevisiae* | France | Champagne | 101 | 55 | 1 | 1 | 3 | 1 | 1 |
| Ψ:Pseudogene. | | | | | | | | | | |
| *The last five nucleotides of these allele sequences were absent, and hence, they were considered as unknown positions in the MJ network analysis: They are located in a different position in the network, for this reason allele 66 appears twice in figure 1A. | | | | | | | | | | |
