# Supplementary material for: Reconstruction of the Evolutionary History of Saccharomyces cerevisiae x S. kudriavzevii Hybrids Based on Multilocus Sequence Analysis
Source: PLoS One. 2012 Sep 25;7(9):e45527. doi: 10.1371/journal.pone.0045527 (PMC3458055; doi:10.1371/journal.pone.0045527)
Supplement: Table S3 — Geographic origin and genetic constitution of Saccharomyces cerevisiae wine strains isolated from different countries [8] . (DOCX) [file pone.0045527.s006.docx]

| **Table S3.** Geographic origin and genetic constitution of *Saccharomyces cerevisiae* wine strains isolated from different countries [8] | | | | | | |
| --- | --- | --- | --- | --- | --- | --- |
| **Strain** | **Country** |  | **Alleles** | | | |
|  |  |  | ***BRE5*** | ***CAT8*** | ***EGT2*** | ***GAL4*** |
| NPCC1155 (9Arg) | Argentina |  | 32 | 33 | 4 | 1 |
| NPCC1157 (10Arg) | Argentina |  | 41 | 31 | 3 | 27 |
| NPCC1161 (29Arg) | Argentina |  | 41 | 82 | 3 | 27 |
| NPCC1162 (30Arg)^1^ | Argentina |  | 32, 41 | 31 | 3 | 27 |
| NPCC1164 (31Arg) | Argentina |  | 32 | 83 | 3 | 27 |
| NPCC1177 (32Arg) | Argentina |  | 32 | 33 | 7 | 77 |
| NPCC1193 (26Arg) | Argentina |  | 41 | 33 | 3 | 27 |
| NPCC1201 (20Arg) | Argentina |  | 41 | 33 | 3 | 27 |
| NPCC1212 (18Arg) | Argentina |  | 31 | 33 | 5 | 27 |
| NPCC1221 (15Arg) | Argentina |  | 32 | 33 | 3 | 27 |
| NPCC1234 (34Arg) | Argentina |  | 32 | 84 | 3 | 27 |
| NPCC1236 (16Arg) | Argentina |  | 32 | 31 | 3 | 27 |
| NPCC1238 (33Arg) | Argentina |  | 31 | 31 | 3 | 27 |
| NPCC1334 (7Arg) | Argentina |  | 41 | 33 | 2 | 27 |
| L962 | Argentina |  | 43 | 32 | 8 | 27 |
| L981 | Argentina |  | 32 | 32 | 3 | 27 |
| L982 | Argentina |  | 41 | 33 | 18 | 27 |
| L1006 | Argentina |  | 41 | 33 | 3 | 85 |
| L16 | Chile |  | 32 | 85 | 3 | 27 |
| L20 | Chile |  | 32 | 33 | 3 | 27 |
| L21 | Chile |  | 32 | 86 | 3 | 27 |
| L146 | Chile |  | 32 | 87 | 3 | 27 |
| L165 | Chile |  | 32 | 32 | 3 | 27 |
| L169 | Chile |  | 41 | 32 | 17 | 27 |
| L246 | Chile |  | 32 | 33 | 3 | 27 |
| L269 | Chile |  | 89 | 31 | 3 | 27 |
| L720 | Chile |  | 91 | 32 | 3 | 27 |
| L1054 | Chile |  | 32 | 33 | 3 | 1 |
| L1374 | Chile |  | 41 | 31 | 3 | 27 |
| ZA13 | South Africa |  | 82 | 33 | 3 | 27 |
| ZA14 | South Africa |  | 27 | 33 | 3 | 1 |
| ZA20^1^ | South Africa |  | 5, 83 | 78 | 11, 50 | 72 |
| ZA26^1^ | South Africa |  | 85, 86 | 5 | 3, 11 | 3 |
| ZA29 | South Africa |  | 87 | 32 | 8 | 27 |
| HA1827 | Austria |  | 31 | 33 | 8 | 27 |
| HA1856 | Austria |  | 42 | 41 | 3 | 1 |
| HA1870 | Austria |  | 41 | 42 | 8 | 27 |
| HA1917 | Austria |  | 43 | 32 | 8 | 27 |
| HA1922 | Austria |  | 34 | 34 | 3 | 27 |
| HA1923 | Austria |  | 32 | 33 | 3 | 27 |
| HA1930 | Austria |  | 32 | 35 | 3 | 27 |
| HA1936 | Austria |  | 35 | 36 | 3 | 32 |
| ZIM1366 | Slovenia |  | 27 | 30 | 3 | 27 |
| ZIM1568 | Slovenia |  | 28 | 31 | 3 | 27 |
| ZIM1914 | Slovenia |  | 30 | 32 | 3 | 27 |
| ZIM2171 | Slovenia |  | 33 | 31 | 10 | 27 |
| CBS1399 | Switzerland |  | 41 | 31 | 3 | 27 |
| CBS2897^1^ | Switzerland |  | 74 | 5 | 11, 15 | 3 |

| **Table S3.** Cont**.** | | | | | | |  |
| --- | --- | --- | --- | --- | --- | --- | --- |
| **Strain** | **Country** |  | **Alleles** | | | |  |
|  |  |  | ***BRE5*** | ***CAT8*** | ***EGT2*** | ***GAL4*** | |
| CECT1477 | France |  | 41 | 31 | 21 | 27 | |
| CECT10692 | France |  | 66 | 55 | 3 | 1 | |
| ICV28 | France |  | 66 | 33 | 3 | 27 | |
| L1323 | France |  | 32 | 89 | 3 | 27 | |
| L1324 | France |  | 32 | 33 | 3 | 27 | |
| L1325 | France |  | 41 | 52 | 3 | 27 | |
| L1329 | Spain |  | 32 | 33 | 3 | 79 | |
| L1330 | France |  | 41 | 55 | 3 | 1 | |
| L1334 | France |  | 41 | 33 | 3 | 27 | |
| L1335 | France |  | 93 | 33 | 3 | 1 | |
| L1338 | France |  | 94 | 33 | 3 | 1 | |
| CETC1475 | Spain |  | 63 | 55 | 3 | 1 | |
| CECT1883 | Spain |  | 64 | 56 | 3 | 27 | |
| CECT10557 | Spain |  | 32 | 61 | 3 | 27 | |
| CECT11757 | Spain |  | 63 | 57 | 3 | 1 | |
| CECT11834 | Spain |  | 68 | 55 | 3 | 1 | |
| CECT12738 | Spain |  | 72 | 46 | 3 | 27 | |

1. Underlined references are for non wine alleles.
2. Heterozygous strain for wine alleles
3. Heterozygous strain for wine and non-wine alleles
