# Supplementary material for: Reconstruction of the Evolutionary History of Saccharomyces cerevisiae x S. kudriavzevii Hybrids Based on Multilocus Sequence Analysis
Source: PLoS One. 2012 Sep 25;7(9):e45527. doi: 10.1371/journal.pone.0045527 (PMC3458055; doi:10.1371/journal.pone.0045527)
Supplement: Table S4 — Geographic origin, source of isolation and genetic constitution of Saccharomyces cerevisiae strains isolated from sources different from wine [8] . (DOCX) [file pone.0045527.s007.docx]

| **Table S4.** Geographic origin, source of isolation and genetic constitution of *Saccharomyces cerevisiae* strains isolated from sources different from wine [8]. | | | | | | | | | |  |
| --- | --- | --- | --- | --- | --- | --- | --- | --- | --- | --- |
| **Strain** | **Country** | **Isolation source** |  | **Alleles^a^** | | | | |  |  |
|  |  |  |  | ***BRE5*** | ***CAT8*** | | ***EGT2*** | ***GAL4*** |  |  |
| CBS8857^2^ | Burkina Faso | Sorgo beer |  | 4, 5 | 5 | | 3, 11 | 3 |  |  |
| CECT10691 | Côte d’Ivore | Palm wine |  | 8 | 62 | | 13 | 56 |  |  |
| CECT11837 | West Africa | Bili wine |  | 8 | 62 | | 13 | 3 |  |  |
| NCAIM Y925^1^ | Hungary | Peach |  | 40 | 27, 40 | | 1 | 34 |  |  |
| CECT10120 | Spain | Bush |  | 41 | 58 | | 3 | 55 |  |  |
| CBS2992 | Pakistan | Palm wine |  | 53 | 50 | | 22 | 45 |  |  |
| CBS1460 | Indonesia | Ferment |  | 58 | 33 | | 3 | 27 |  |  |
| CBS1201^1^ | Japan | Sorgo |  | 77 | 70, 71 | | 25 | 63, 64 |  |  |
| CBS1198 | Japan | Sake |  | 9 | 21 | | 11 | 62 |  |  |
| CBS1199 | Japan | Sake |  | 9 | 21 | | 11 | 57 |  |  |
| CBS6412 | Japan | Sake |  | 79 | 21 | | 11 | 67 |  |  |
| CBS6414 | Japan | Sake |  | 79 | 74 | | 11 | 68 |  |  |
| CECT10711 | Japan | Sake |  | 9 | 21 | | 11 | 57 |  |  |
| UFMG-A2426 | Brasil | Cachaça |  | 32 | 39 | | 3 | 27 |  |  |
| GU4^2^ | Mexico | Tequila |  | 23, 24 | 25, 26 | | 11 | 25, 26 |  |  |
| PE30C^1^ | Peru | Chicha |  | 5, 7 | 3 | | 13 | 13 |  |  |
| PE7M^2^ | Peru | Masato |  | 5 | 3 | | 3, 29 | 6, 7 |  |  |
| PE94M^2^ | Peru | Masato |  | 4, 5 | 5 | | 3, 11 | 3 |  |  |
| PE103M^2^ | Peru | Masato |  | 5 | 3 | | 3, 29 | 13, 14 |  |  |
| a- Underlined haplotyopes indicate non wine alleles. | | | | | |  | | |  |  |
| ^1^ Heterozygous strain for non-wine alleles. | | | | | |  | | |  |  |
| ^2^ Heterozygous strain for wine and non wine alleles. | | | | | |  | | |  |  |
