# Supplementary material for: Reconstruction of the Evolutionary History of Saccharomyces cerevisiae x S. kudriavzevii Hybrids Based on Multilocus Sequence Analysis
Source: PLoS One. 2012 Sep 25;7(9):e45527. doi: 10.1371/journal.pone.0045527 (PMC3458055; doi:10.1371/journal.pone.0045527)
Supplement: Table S5 — PCR primers designed in the present study to amplify five nuclear gene regions. PCR primers designed in the present study to amplify five nuclear gene regions. Those primers labeled with K and C are specific primers for S. kudriavzevii and S. cerevisiae alleles, respectively. (DOCX) [file pone.0045527.s008.docx]

**Table S5.** PCR primers designed in the present study to amplify five nuclear gene regions. Those primers labeled with K and C are specific primers for *S. kudriavzevii* and *S. cerevisiae* alleles, respectively.

| **Primer** | **Sequence** |
| --- | --- |
| CYR1_3K (reverse) | 5’-TTggATTTTCTggAATgTTCTCATTAggCCgC-3’ |
| CYR1_3C (reverse) | 5’-TCAgAgTTAgATTTTCCggAATgTTCTCATTATAT-3’ |
| CYC3_5K (forward) | 5’-TCTCCgCAgATTAACCCCggTCAgCAggTg-3’ |
| CYC3_5C (forward) | 5’-CgggCAAAgATATTggTggggCAgCAgTA-3’ |
| CYC3_3 (reverse) | 5’-gggAACAgTAggCCgCACARRTgCATCCA-3’ |
| BRE5_5 (forward) | 5’-TgATTATAgCCACgggTgARATgTTYTgg-3’ |
| BRE5_3K (reverse) | 5’-TTCgCAACCggTTCTAAAgAgggCgAAAC-3’ |
| BRE5_3C (reverse) | 5’-gAAgATgAAggTgTTgAAgCgTTATTgCC-3’ |
| CAT8a_5 (forward) | 5’-AAgAgCAACTATAgYCTgACAAARYTAATgAg-3’ |
| CAT8Ka_5 (forward) | 5’-CCATCCTGAGGAACCAAATTGC-3’ |
| GAL4_5 (forward) | 5’-TgTgCCAAgTgTCTgAAgAAYAAYTgggA-3’ |
| GAL4_3 (reverse) | 5’-gCgATTTCAATCTgATTATTRTACARCATCAT-3’ |
| GAL4Ka_5 (forward)^+^ | 5’-GAAGCTGTTGTCTTCAATGG-3’ |
| GAL4Ka_3 (reverse)^+^ | 5’-CTTGTATTTGGTTTCTGTCTCC-3’ |
| EGT2n_3 (reverse)* | 5’-CCAggCggTRTTATTAgTTTTgTATATRCCACC-3’ |
| EGT2n_5 (forward)* | 5’-CAgATCATTggTTCATAATAgAAggKCAAYTgT-3’ |
| EGT2Ka_5 (forward) | 5’-ACACACGCTCTTACACAAACGCAG-3’ |
| EGT2Ka_3 (reverse) | 5’-TTAGTGGTGGAGCCGACATTAGCA-3’ |

*Annealing Temperature = 50ºC

^+^GAL4_5 and GAL4_3 were used for sequencing PCR fragments amplified with GAL4Ka_5 and GAL4Ka_3
