# Supplementary material for: Reconstruction of the Evolutionary History of Saccharomyces cerevisiae x S. kudriavzevii Hybrids Based on Multilocus Sequence Analysis
Source: PLoS One. 2012 Sep 25;7(9):e45527. doi: 10.1371/journal.pone.0045527 (PMC3458055; doi:10.1371/journal.pone.0045527)
Supplement: Table S6 — Summarized results on the putative origin of hybrids based on the hybrid allele groups defined according to Maximum-Parsimony (Figure S2), and Neighbor-Joining (Figure S3) phylogenetic analyses of gene sequences. Allele groups are highlighted in the same colors used to indicate allele groups in the Maximum-Parsimony and Neighbor-Joining gene trees depicted in Figures S2 and S3, respectively. Symbols: −, gene lost in the hybrid; 0, no group differentiation. (DOCX) [file pone.0045527.s009.docx]

**Table S6.** Summarized results on the putative origin of hybrids based on the hybrid allele groups defined according to Maximum-Parsimony (Figure S2), and Neighbor-Joining (Figure S3) phylogenetic analyses of gene sequences. Allele subgroups are highlighted in the same colors used to indicate allele groups in the Maximum-Parsimony and Neighbor-Joining gene trees depicted in Figures S2 and S3, respectively. Symbols: −, gene lost in the hybrid; 0, no group differentiation.

|  |  | ***S. cerevisiae* subgroups** | | | | | | | |  | ***S. kudriavzevii* subgroups** | | | | | | | |  | **Hybrid^1^**  **Combinations** |
| --- | --- | --- | --- | --- | --- | --- | --- | --- | --- | --- | --- | --- | --- | --- | --- | --- | --- | --- | --- | --- |
| **Strain** |  | ***BRE5*** | ***CAT8*** | ***CYC3*** | ***CYR1*** | ***EGT2*** | ***GAL4*** | ***MET6*** | **Genotype** |  | ***BRE5*** | ***CAT8*** | ***CYC3*** | ***CYR1*** | ***EGT2*** | ***GAL4*** | ***MET6*** | **Genotype** |  |  |
| **HA1835** |  | **C1** | **C1** | **0** | **0** | **0** | **C1** | **C1** | **CG1** |  | **K1** | **K1** | **K1** | **K1** | **0** | **0** | **0** | **KG1** |  | **1** |
| **HA1837** |  | **C1** | **C1** | **0** | **0** | **0** | **C1** | **C1** | **CG1** |  | **K1** | **K1** | **K1** | **K1** | **0** | **0** | **0** | **KG1** |  | **1** |
| **HA1841** |  | **C1** | **C1** | **0** | **0** | **0** | **C1** | **C1** | **CG1** |  | **K1** | **K1** | **K1** | **K1** | **0** | **0** | **0** | **KG1** |  | **1** |
| **HA1842** |  | **C1** | **C1** | **0** | **0** | **0** | **C1** | **C1** | **CG1** |  | **K1** | **K1** | **K1** | **K1** | **0** | **0** | **0** | **KG1** |  | **1** |
| **VIN7** |  | **C1** | **C1** | **0** | **0** | **0** | **C1** | **C1** | **CG1** |  | **K1** | **K1** | **K1** | **K1** | **0** | **0** | **0** | **KG1** |  | **1** |
| **SOY3** |  | **C3** | **C1** | **0** | **0** | **0** | **C1** | **C1** | **CG1** |  | **K1** | **K1** | **K1** | **K1** | **0** | **0** | **0** | **KG1** |  | **1** |
| **W27** |  | **C2** | **C2** | **0** | **0** | **0** | **C2** | **C2** | **CG2** |  | **K2** | **K2** | **K2** | **K2** | **0** | **0** | **0** | **KG2** |  | **2** |
| **SPG14-91** |  | **C2** | **C2** | **0** | **0** | **0** | **C2** | **C2** | **CG2** |  | **K2** | **K2** | **K2** | **K2** | **0** | **0** | **0** | **KG2** |  | **2** |
| **SPG16-91** |  | **C2** | **C2** | **0** | **0** | **0** | **C2** | **C2** | **CG2** |  | **K2** | **K2** | **K2** | **K2** | **0** | **0** | **0** | **KG2** |  | **2** |
| **126** |  | **C2** | **C2** | **0** | **0** | **0** | **C2** | **C2** | **CG2** |  | **K2** | **K2** | **K2** | **K2** | **0** | **0** | **0** | **KG2** |  | **2** |
| **172** |  | **C2** | **C2** | **0** | **0** | **0** | **C2** | **C2** | **CG2** |  | **K2** | **K2** | **K2** | **K2** | **0** | **0** | **0** | **KG2** |  | **2** |
| **319** |  | **C2** | **C2** | **0** | **0** | **0** | **C2** | **C2** | **CG2** |  | **K2** | **K2** | **K2** | **K2** | **0** | **0** | **0** | **KG2** |  | **2** |
| **441** |  | **C2** | **C2** | **0** | **0** | **0** | **C2** | **C2** | **CG2** |  | **K2** | **K2** | **-** | **K2** | **0** | **0** | **0** | **KG2** |  | **2** |
| **W46** |  | **C2** | **C2** | **0** | **0** | **0** | **C2** | **C2** | **CG2** |  | **K2** | **K2** | **K2** | **K2** | **0** | **0** | **0** | **KG2** |  | **2** |
| **CECT11003** |  | **C2** | **C2** | **0** | **0** | **0** | **C2** | **C2** | **CG2** |  | **K2** | **K2** | **K2** | **K2** | **0** | **0** | **0** | **KG2** |  | **2** |
| **CECT11004** |  | **C2** | **C2** | **0** | **0** | **0** | **C2** | **C1** | **CG2** |  | **K2** | **K2** | **K2** | **K2** | **0** | **0** | **0** | **KG2** |  | **2** |
| **CECT1990** |  | **C2/2** | **C2** | **0** | **C3** | **0** | **C2** | **C1** | **CG3** |  | **-** | **K1** | **K1** | **K3** | **0** | **0** | **0** | **KG3** |  | **3** |
| **CECT11011** |  | **C2** | **C2** | **0** | **C3** | **0** | **C2/2** | **C1** | **CG3** |  | **K2** | **K1** | **-** | **K3** | **0** | **0** | **0** | **KG3** |  | **3** |
| **CECT1388** |  | **C2/2** | **C2** | **0** | **0** | **0** | **C1** | **C1** | **CG4** |  | **-** | **K1** | **K1** | **K3** | **0** | **0** | **0** | **KG3** |  | **4** |
| **CECT11002** |  | **2** | **C2** | **0** | **0** | **0** | **C1** | **C1** | **CG4** |  | **-** | **-** | **K1** | **K3** | **0** | **0** | **0** | **KG3** |  | **4** |
| **MR25** |  | **C3/3** | **C2** | **0** | **0** | **0** | **C1** | **C1** | **CG5** |  | **-** | **K1** | **-** | **K3** | **0** | **0** | **0** | **KG3** |  | **5** |
| **IF6** |  | **C3** | **C2** | **0** | **0** | **0** | **C1** | **C1** | **CG5** |  | **K1** | **-** | **-** | **K1** | **0** | **0** | **0** | **KG1** |  | **6** |
| **AMH** |  | **C3** | **C2** | **0** | **0** | **0** | **C1** | **C1** | **CG5** |  | **-** | **K1** | **-** | **-** | **-** | **-** | **-** | **KG4** |  | **7** |
| **PB7** |  | **C3** | **C1** | **0** | **0** | **0** | **C2** | **C1** | **CG6** |  | **K2** | **K2** | **K1** | **K1** | **K3** | **0** | **0** | **KG5** |  | **8** |
| **CBS2834** |  | **C1** | **C1** | **0** | **0** | **0** | **C1** | **C1** | **CG1** |  | **K2** | **K1** | **-** | **K1** | **0** | **0** | **0** | **KG6** |  | **9** |
| **CID1** |  | **-** | **C1** | **0** | **0** | **0** | **C2** | **C1** | **CG6** |  | **K2** | **K1** | **K1** | **K1** | **0** | **0** | **0** | **KG6** |  | **10** |

^1^ Hybrid combinations correspond to different combinations of the putative parental genotypes.
