# Supplementary material for: Reconstruction of the Evolutionary History of Saccharomyces cerevisiae x S. kudriavzevii Hybrids Based on Multilocus Sequence Analysis
Source: PLoS One. 2012 Sep 25;7(9):e45527. doi: 10.1371/journal.pone.0045527 (PMC3458055; doi:10.1371/journal.pone.0045527)
Supplement: Table S7 — List of chromosome rearrangements found in the dietary supplement IF6 and the clinical MR25 hybrid strains. (DOCX) [file pone.0045527.s010.docx]

**Table S7.** List of chromosome rearrangements found in the dietary supplement IF6 and the clinical MR25 hybrid strains.

| **Strain** | **Chromo-**  **some** | **Rearrangement** | **Breakpoint interval** | **Putative recombining sequences** |
| --- | --- | --- | --- | --- |
| MR25 | IV | Chimerical chromosome | YDL184C-YDL175C | ARS |
|  | VII | Chimerical chromosome | YGR187C-YGR198W | Ty1 LTR, ARS, tRNA-Lys or tRNA-Trp |
|  | IX | Chimerical chromosome | YIL129C-YIL124W | ARS |
|  | XII | Chimerical chromosome | YLL026C-YLL016W | Ty1 LTR, tRNA-Pro or ARS |
|  | XIV | Chimerical chromosome | YNR029C-YNR032W | ARS |
|  | XtXIII | *S. kudriavzevii* hypothetical non-reciprocal translocation |  |  |
| IF6 | X | Chimerical chromosome | YJL108C-YJL101C | ARS |
|  | XII | Chimerical chromosome | YLR257W-YLR249W | Ty1 LTRs, Ty3 LTRSs, tRNA-Ala or ARS |
|  | XIII | Chimerical chromosome | YMR044W-YMR055C | Ty1 LTRs, tRNA-Trp or tRNA-Val |
|  | XII | Segment deletion | YLR155C-YLR256W |  |
|  | VIItIX | *S. kudriavzevii* hypothetical non-reciprocal translocation |  |  |
